# An efficient feature pyramid network with adaptive LSTM for pest detection and classification in IoT

**DOI:** 10.1038/s41598-025-34409-1

**Published:** 2026-01-06

**Authors:** Rajasekaran Arunachalam, Mohana Jaishankar, Amit Arora, Padmapriya Shanmugam, Sumanth Venugopal, Thella Preethi Priyanka

**Affiliations:** 1 Department of Electronics and Communication Engineering, Saveetha School of Engineering, Saveetha Institute of Medical and Technical Sciences, Thandalam, Chennai, Tamil Nadu 602105 India; 2 Department of Electrical, Electronics and Communication Engineering, Galgotias University, Greater Noida, Uttar Pradesh 203201 India; 3 Department of Artificial Intelligence and Data Science, Velammal Institute of Technology, Ponneri, Tamil Nadu 601204 India; 4 Manipal Institute of Technology Bengaluru, Manipal Academy of Higher Education, Manipal, India; 5 Centre for Research Impact & Outcome, Chitkara University Institute of Engineering and Technology, Rajpura, Punjab 140401 India

**Keywords:** Pest detection and classification, Multi-attention fusion vision transformer-based adaptive long short-term memory, Enhanced and intelligent gooseneck barnacle optimization with randomized exploration, Internet of things, Biological techniques, Computational biology and bioinformatics, Engineering

## Abstract

Crop pests are a major cause of economic loss and environmental damage globally. Timely detection of pests is crucial for protecting crops and maintaining the global food supply. However, existing diagnostic methods are especially manual, demanding significant time and expert knowledge. Incorrect pest identification can result in the misuse of pesticides, affecting both crop yields and the surrounding ecosystem. Therefore, there is a need for an automated solution that offers more precise pest identification and classification. So, in this research work, a new Internet of Things (IoT)-based pest detection and classification technique is implemented. In the initial phase, essential images are collected from a standard database that includes the IoT sensor-based pest images. Next, the IoT sensor-based images are offered as the input to the Joint pest detection and classification phase. In this phase, a new framework named Feature Pyramid Network with Multi-Attention Fusion Vision Transformer-based Adaptive Long Short Term Memory (FPN-MAFViT-ALSTM) is employed to execute the pest detection and classification procedure. Moreover, parameters in FPN-MAFViT-ALSTM are tuned using Enhanced and Intelligent Gooseneck Barnacle Optimization with Randomized Exploration (EIGBO-RE), which helps in improving pest detection and classification. At last, pest detection and classified outcomes are obtained from FPN-MAFViT-ALSTM, and then various experiments are carried out to verify its efficiency under varying conditions.

## Introduction

Globally, agriculture is the backbone behind the development of economic growth. Resilient agriculture contributes to the growth of developing countries, leading to increased food security^1^. As the crop yields become more productive, numerous benefits emerge, including a decline in the food price and enhanced living standards for humans^2^. However, several factors affect the production of crop yield, including varying weather conditions, pest disease, soil degradation and so on^3^. To maximize the economic scale of the agricultural sector, pest detection and pest disease classification are crucial. IoT is a device with an embedded sensor system that works by collecting and exchanging data over the network^4^. Moreover, IoT focuses on introducing artificial intelligence in the pest control system^5^. By placing effective IoT network sensors on the agricultural field, the pest control system analyzes the leaf, soil moisture, humidity and structure of plants that might indicate the presence of pests^6^.

Furthermore, pest control methods for analyzing pests of varying sizes are essential to implement an accurate pest identification model^7^. Despite that, insect classification is a complex task due to the similarities between different pests^8^. Many pest detection monitoring system arises, which are time-consuming and contain challenging processes requiring specialized knowledge^9^. The manual approaches are more computationally complex, leading to misclassification. Moreover, traditional machine learning algorithms have some limitations. These techniques face difficulties, including limited pest species, leading to underperformance^10^. Hence, it requires a feature extraction process that is essential for accurate classification. Feature selection faces a significant challenge in detecting pests^11^. A variation in pest sizes in plants, with different structures and sizes makes the distinct feature selection vectors difficult^12^. Therefore, an advanced pest detection strategy is essential for pest recognition and to determine pest severity, making the model achieve high results^13^.

Nowadays, deep learning plays a vital role in automatically detecting and classifying pests in agriculture. Many pest classification techniques using deep learning have been developed^14^. Deep Learning algorithms, like Deep Neural Networks (DNNs), are also implemented to understand complex patterns and automatically extract features^15^. DNNs are good at classifying objects by training the samples faster and more efficiently. These methods use transfer learning, where models are already trained to perform new tasks^16^. Deep learning methods commonly use Convolutional Neural Networks (CNNs) to identify important characteristics from data not require expert knowledge^17^. Due to these advancements, deep learning methods are widely used to solve complex problems. Therefore, in this study, an advanced deep learning model was designed aimed at improving generalization during real-time crop pest detection.

Motivation: In present days, more research work has been designed to perform the pest detection and classification procedures. Machine learning and deep learning are widely used techniques for detecting pests in the agricultural field. Among all the deep learning techniques, CNN has received a huge attention from researchers, and the variants of CNN also provided better outcomes in wide classes. ResNeXt CNN^34^ is an efficient technique employed to identify the fungal diseases in the plants, and it has the ability to classify into 3 different classes. Next, a unique technique is designed by considering Artificial Intelligence with Computer Vision (AI-CV)^35^ to find various diseases that affect the crop and also improve the overall pest detection efficiency. ResNet-50^36^ is a novel mechanism for identifying pests in the crops, and this mechanism includes the segmentation procedure to identify the specific disease-affected regions. Furthermore, meta-learning models^37^ are introduced to identify the pests in plants through scalable parameters. Here, refining classifiers are used in the metric space that uses the cosine distance for predicting the pests. Moreover, the fused combination of CNN techniques like Inception-v7 with ResNet^38^ is used for predicting the pests. This combination accomplished superior outcomes in detecting the pest at the early stages. Yet, the existing pest detection techniques use the required images from the benchmark resources, which offer minimal accuracy in detecting the pest under different classes. In some cases, they lead to overlapping problems that impact pest classification efficiency. In order to attain higher quality pest detection outcomes, a novel IoT-based pest detection and classification framework is designed to accomplish comparatively higher performance in complex classes.

Contributions: The important contributions are described below.

- To implement an effective deep learning approach with IoT for early detection and classification of pests that helps to reduce crop loss by identifying the pests in the early stages. The integrated deep learning with IoT mechanism is helpful to enable more effective agricultural practices by optimizing the usage of pesticides. The developed model is trained using a diverse set of labeled pest images, which ensures the robust performance across different crop types, growth stages and environmental conditions.
- To present a joint pest detection and classification model using FPN-MAFViT-ALSTM for providing highly adaptable and accurate solutions for pest management. The developed FPN-MAFViT-ALSTM model integrates a pyramid network, multi-attention fusion and a ViT-based LSTM architecture, which is effective for continuously analyzing the contextual parameters to provide accurate pest detection results. The varying-sized pests are effectively identified through the FPN module and the multi-attention fusion module dynamically prioritizes the relevant features to effectively identify the pest characteristics. The ViT component effectively captures the global contextual relationships from the images, which provides more suitable results over complex backgrounds. The adaptive LSTM component effectively analyzes the temporal data, which enhances the ability of the system to adapt to varying environmental conditions.
- To develop an optimization algorithm, EIGBO-RE by updating the traditional Gooseneck Barnacle Optimization Algorithm (GBOA) to provide the most effective solutions for the complex optimization issues. The pest detection and classification performance is greatly improved by iteratively optimizing the parameters such as hidden neurons and learning rate in FPN and LSTM during network training. The position of the EIGBO-RE algorithm is updated based on the fitness of the members, which increases the convergence performance and optimal solution searching performance in the problem space.

Organization: The overall model organization is outlined in this section. In “Literature survey” literature survey of prior pest detection techniques is provided. The significance of IoT and the rationale of the deep learning model in the agricultural field are given in “IoT in agricultural field for pest detection and classification”. “Overall description of developed system and parameters tuning model” includes the overall network structure with enhanced optimization. “Pest detection and classification using multi-attention fusion and adaptive network” characterized the designed pest detection and classification mechanism. “Results and discussion” illustrates the result and discussion, and the conclusion is available in “Conclusion”.

## Literature survey

In this section, various existing research works employed to execute the pest detection and classification process are discussed in “Related works”. Moreover, multiple issues take place in the prior pest detection and classification mechanism is elaborated in “Problem statement” with their advancement and complications.

### Related works

In 2023, Ali et al.^18^ have proposed a new hybrid deep-learning technique, Faster-Recurrent Convolutional Neural Network (Faster-RCNN) with MobileNet was trained to analyze and recognize different pest species. Furthermore, the robustness of the Faster-RCNN with MobileNet was evaluated on a well-trained approach. Moreover, the performance indicators demonstrated the robustness and reliability of novel deep-learning-based approach.

In 2019, Liu et al.^19^ developed a novel deep learning technique PestNet, which can detect and classify pest insects. Here, Channel-Spatial Attention (CSA) was employed with CNN to extract essential features. In the second phase, a Region Proposal Network (RPN) was utilized to generate regional recommendations about the possible insect positions. Thirdly, a Position-Sensitive Score Map (PSSM) was employed to enhance the pest detection process. The outcomes indicated that the developed PestNet outperformed better than the conventional model.

In 2024, Karthik et al.^20^ have created an advanced feature fusion model by using bi-concurrent tracks. Two custom blocks were utilized namely Multi-Separable Attention (MSA) and Tri Shuffle Convolution Attention (TSCA) blocks. By leveraging the strength of Dual-Attention Multi-scale Fusion Networks (DAMFN), the developed approach provided accurate detection outcomes.

In 2024, Vilar-Andreu et al.^21^ have designed a novel approach for pest disease detection by implementing a YOLOv8-based tool. Its primary objective was to identify and detect pests in the crop instantly. The distinct phases of the model’s performance during the training process were evaluated under the mAP50 value. Additionally, this novel approach incorporated essential principles that expanded the potential of YOLOv8, enabling the model to identify the presence of pests even in large datasets.

In 2023, Prasath and Akila^22^ have introduced an innovative strategy for precise pest detection and classification namely the Yolov3 model, through a classifier and object detection process. To optimize the accuracy of the model, the Adaptive Energy-based Harris Hawks Optimization (AE-HHO) algorithm was employed. At last, the outcomes of the developed model were evaluated under different performance metrics.

In 2024, Hasan et al.^23^ have established an IoT-enabled solution for tomato farming and pest management approach by leveraging a deep learning strategy, sensors and image recognition. The integration of IoT sensors and cameras helped to collect images. Then, the developed models were tested in MobileNet and DenseNet201. Moreover, this model was trained to categorize different species.

In 2024, Ahmed et al.^24^ have developed a new IoT-aided pest management system to detect pests with embedded computing. The developed model employed a trained CNN, from which colour histogram features were extracted. The developed system demonstrated a recall score of 86.2%, highlighting the accurate classification of disease.

In 2024, Kathole et al.^25^ have created a model for pest detection and classification. Initially, data were collected through IoT devices. The acquired images were then analyzed using object detection with YOLOv3. To optimize the classifier’s parameters, the Adaptive Honey Badger Algorithm (AHBA) was employed. The obtained results indicated that the suggested approach was advantageous, as it enhanced the efficiency of agricultural data collection and ensured robust technical support.

### Problem statement

Pest detection and recognition play a main role in maintaining the health and ecological balance and boosting food production. A sudden, unusual increase in pests and insects significantly impacts agricultural fields. Existing systems rely on the IoT sensors to collect images for detecting and classifying the pests, but these methods do not yield higher accuracy. Existing models that are being used for accurate detection and classification of pest are shown in Table 1.

- In existing works, holistic features present in pest images are being captured insufficiently in manual feature extraction; because of this, it automatically reduces the accuracy and adaptability of the detection algorithm. Therefore, it is necessary to use an automatic feature extraction strategy that can be built into the classification technique.
- Convolutional machine learning-based pest detection and identification techniques typically depend on manually created feature extraction methods, which demand a considerable amount of expertise and experience. So, to overcome these problems, planned to design a deep learning model for precise pest identification and classification.
- Deep learning offers a powerful approach for both detecting and classifying pests in agricultural settings, enabling faster and more accurate pest identification and management. But it requires large, labeled datasets, which can be difficult and expensive to collect. The performance of deep learning models can be sensitive to data quality, such as noise, blur, and illumination variations.
- As crop pest images are typically captured in natural agricultural settings, leading to complex backgrounds and variable lighting, this potentially minimizing the accuracy rate of conventional algorithms. To overcome this problem, a new technology called the vision transformer technique can be used, and it uses pre-trained deep learning models, which were trained on extensive datasets and adapts them for pest detection tasks.
- Pest detection and classification without optimization often suffer from class imbalance, overfitting, and slow processing. These issues lead to poor model performance, including false detections and inefficient real-time applications. Advanced optimization algorithms can be used to improve accuracy, balance data, and speed up inference. By fine-tuning model parameters, overall detection efficiency and reliability are significantly enhanced. Hence, a well-advanced deep-learning mechanism is developed for pest detection and classification.

**Table 1:** Merits and demerits of prior pest detection and classification schemes.

Author [citation]	Methods	Pros	Cons	Key features of the developed model
Ali et al.^18^	Faster-PestNet	It has enhanced speed and effectiveness in pest detection It has been accurate and effectively adapted to perform a range of agricultural tasks	It consumes more time for data labeling It requires expert knowledge for training, tuning, and deployment	Time complexity issues takes place in the network are tackled by considering EIGBO-RE, which reduces the complication by tuning the parameters in the training phase
Liu et al.^19^	PestNet	It can detect multiple pests simultaneously It is highly adaptable to various agricultural environments	It has more computational time which may affect real-time performance It requires large labeled datasets for effective pest detection	This is issues in tackled by training the network using a novel optimization scheme that supports using the most significant parameter for validation
Karthik et al.^20^	Dual-Attention Multi-scale Fusion Network (DAMFN)	It has enhanced accuracy and precision in disease classification It works well in real-world agricultural environments with pests of different scales	Poor or imbalanced data can cause overfitting It has a higher computational complexity	These issues are tackled by considering high quality dataset for the validation, which collects the required images with the help of IoT sensors
Vilar-Andreu et al.^21^	YOLO	It enables precise and quick decision-making in crops It has excellent performance even with dense or cluttered backgrounds	Network efficiency in complex classes need to be improved	The designed technique is better in obtaining better outcomes by tackling the convergence problems and also obtains the optimal solutions through EIGBO-RE
Prasath B and M. Akila^22^	Yolov3	It is designed for high-speed detection and is ideal for real-time pest monitoring It can detect multiple pest species in one image	It needs a large labeled dataset of pests for accurate detection It has a fixed number of classes	In this research work, accurate pest detection and classification are obtained through FPN-MAFViT-ALSTM. Here, robustness and accuracy are improved through LSTM and FPN
Hasan et al.^23^	MobileNet	It has low latency and faster inference It is lightweight and optimized for mobiles or low-powered devices	Its performance heavily depends on high-quality and well-processed data It tends to underperform in detecting small objects, especially when combined with basic detectors	These issues are tackled by using the ViT, which is good at acquiring the longer range dependencies in wide classes. Moreover, the multi-attention function process in the network supports to acquire the most significant features
Ahmed et al.^24^	CNN	It has automatic feature extraction and no need for manual feature engineering It provides cost-effective systems and deploys reliable for the autonomous recognition of pests	It was unable to maintain precision when counting detected pests in a complex image It consumes more time due to the complexity of the algorithm	Here, a novel optimization model named EIGBO-RE, which supports tuning the parameters precisely and also reduces the errors while classification processes are carried out
Kathole et al.^25^	Adaptive honey badger algorithm	It facilitated precise and reliable pest identification in farming environments It has effective image segmentation and classification	It is not ideal for real-time detection or low-powered devices It requires high-quality labelled images or sensor data for effective pest detection	In order to rectify the complications, the implemented scheme uses the images from benchmark database that use the IoT sensors to collect the images, which supports to use with the low-powered devices

## IoT in agricultural field for pest detection and classification

This section discusses the significance of IoT in the agricultural field in “Significance of IoT in the agricultural field”. The need for choosing a deep learning is provided in “Rationale on deep learning for pest detection and classification”. Used dataset details given in “Experimental image collection”.

### Significance of IoT in the agricultural field

**Fig. 1:**
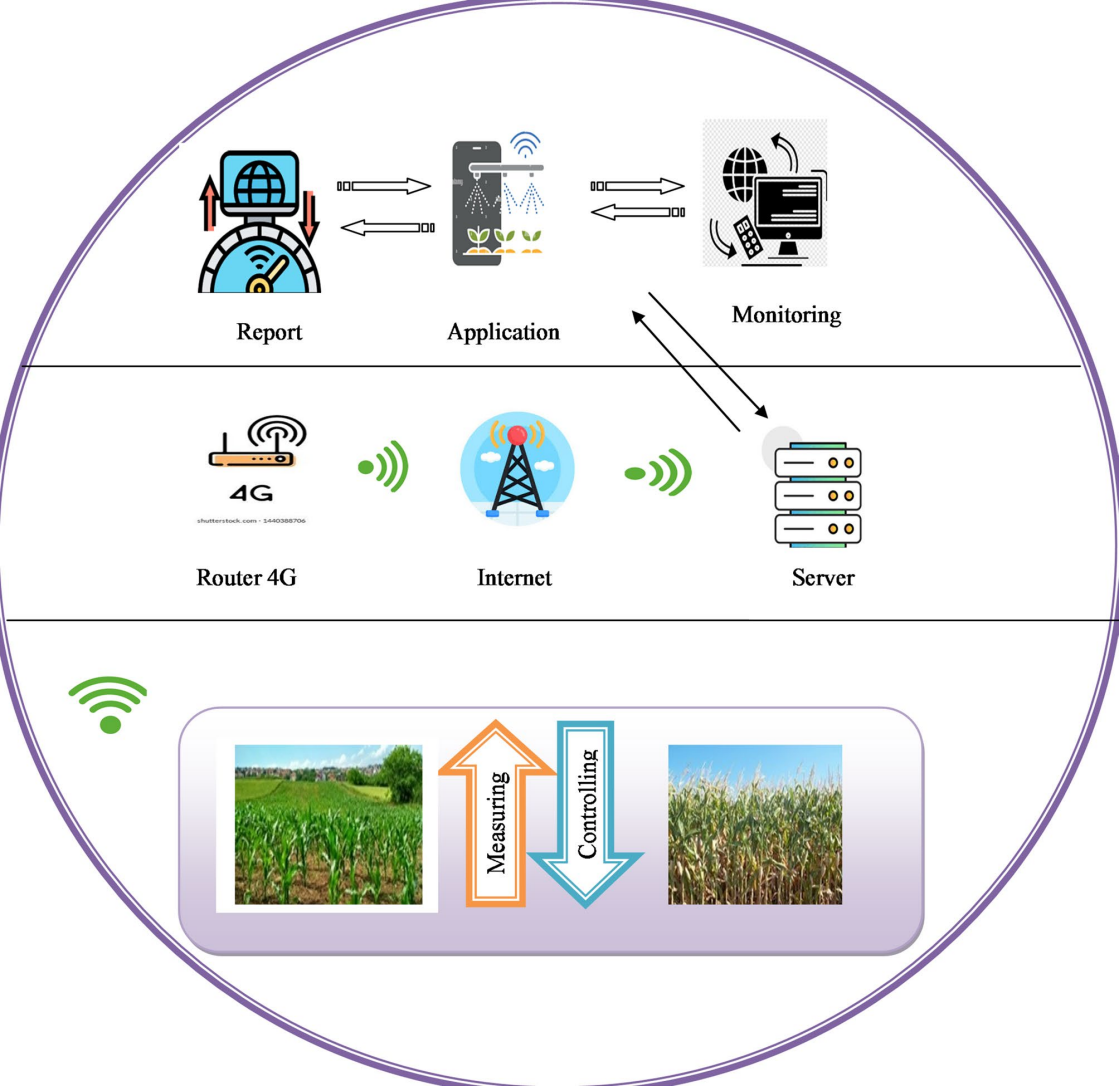
Basic steps in IoT-based agricultural system.

IoT in the agricultural field plays a critical role in enabling precision farming by effectively utilizing resources to help decrease the impacts of environmental practices. Commonly, in agricultural practices, computer vision-based techniques may increase input costs when dealing with complex problems. To tackle these issues, IoT sensors have been introduced as a solution. By reducing computational cost, the IoT sensors enable real-time processing and analysis of agricultural data, thereby minimizing latency and enhancing crop production. However, IoT sensors can automatically collect input images from standard datasets. By collecting real-time data from the available resources, IoT sensors help the detection models to process the data, thereby facilitating accurate outcomes. Therefore, much industrial management has transferred to IoT systems for digital farming to enhance the global market. Figure 1 shows the general architecture of IoT in the agricultural field.

### Rationale on deep learning for pest detection and classification

Globally, crop quality and productivity are impacted by insect pests. Commonly used pest monitoring schemes are based on manual observation and light-based traps, which often lack accuracy and efficiency, resulting in limited real-time responsiveness and the need for large-scale adoption. Additionally, these techniques are insufficient for adapting to the dynamic behavior of pests and the complexity of agricultural ecosystems. Moreover, manual feature extraction makes the model insufficient for capturing the detailed characteristics of pest images, which minimizes the precision and generalizability of traditional systems. To address these challenges, advanced deep learning techniques have been introduced into the agricultural sector to enhance crop development conditions and boost agricultural productivity. Deep learning-based pest detection models have demonstrated superior performance, even in identifying small-sized pest species, enhancing the accuracy of detection in complex agricultural settings. Thus, incorporating deep learning techniques has significantly contributed to early pest identification, reducing crop damage and boosting yield, thereby supporting a steady increase in economic value.

### Experimental image collection

The pest images from the IoT-based system were obtained from the website listed below to identify and classify pests.

Dataset-1 (“Pest Dataset”): The website used to retrieve the pest image is https://www.kaggle.com/datasets/simranvolunesia/pest-dataset. Access data: 2025-04-21. This file is established with a total size of 73.25 MB. It includes 3150 files with related labels. This dataset classifies crop types, offering valuable information for accurate pest recognition tasks.

Dataset-2 (“Agricultural Pest Dataset”): The images are sourced via the website https://www.kaggle.com/datasets/gauravduttakiit/agricultural-pests-dataset. Access data: 2025-04-21. Having an overall size of 107.84 MB, the dataset comprises 5,496 files, with pest images sourced from Flickr. The primary pest classes represented in this dataset include wasps, snails, and caterpillars.

Dataset-3 (“IP102-Dataset”): The images were obtained from https://www.kaggle.com/datasets/rtlmhjbn/ip02-dataset. Access date: 2025-04-21. With a size of 3.19 GB, this file consists of information about 102 classes of pests, including rice leaf roller, yellow cutwork, red spider, rice shell pest and so on. The dataset was divided in the range of 6:1:3. This dataset for pest detection provides sustainable information about the pest.

The gathered IoT pest images are defined by the term $P_T^{img}$.

## Overall description of developed system and parameters tuning model

In this section, a deep discussion about the developed pest detection model is offered in “Description of proposed model”. Novel technique EIGBO-RE employed for parameter tuning is elaborated in “Technical innovations of the proposed approach”, and also the importance of parameter tuning through EIGBO-RE is highlighted in “Developed EIGBO-RE”.

### Description of proposed model

Nowadays, plant disease is a major cause of sustainable damage in crop yield, impacting plant health. Due to the scalability issue and unbalanced data, the classical system performs poorly, leading to inaccurate detection. Thereby, an advanced deep learning approach is developed to tackle the issues faced by the prior models, which is important for detecting and classifying the pests.

**Fig. 2:**
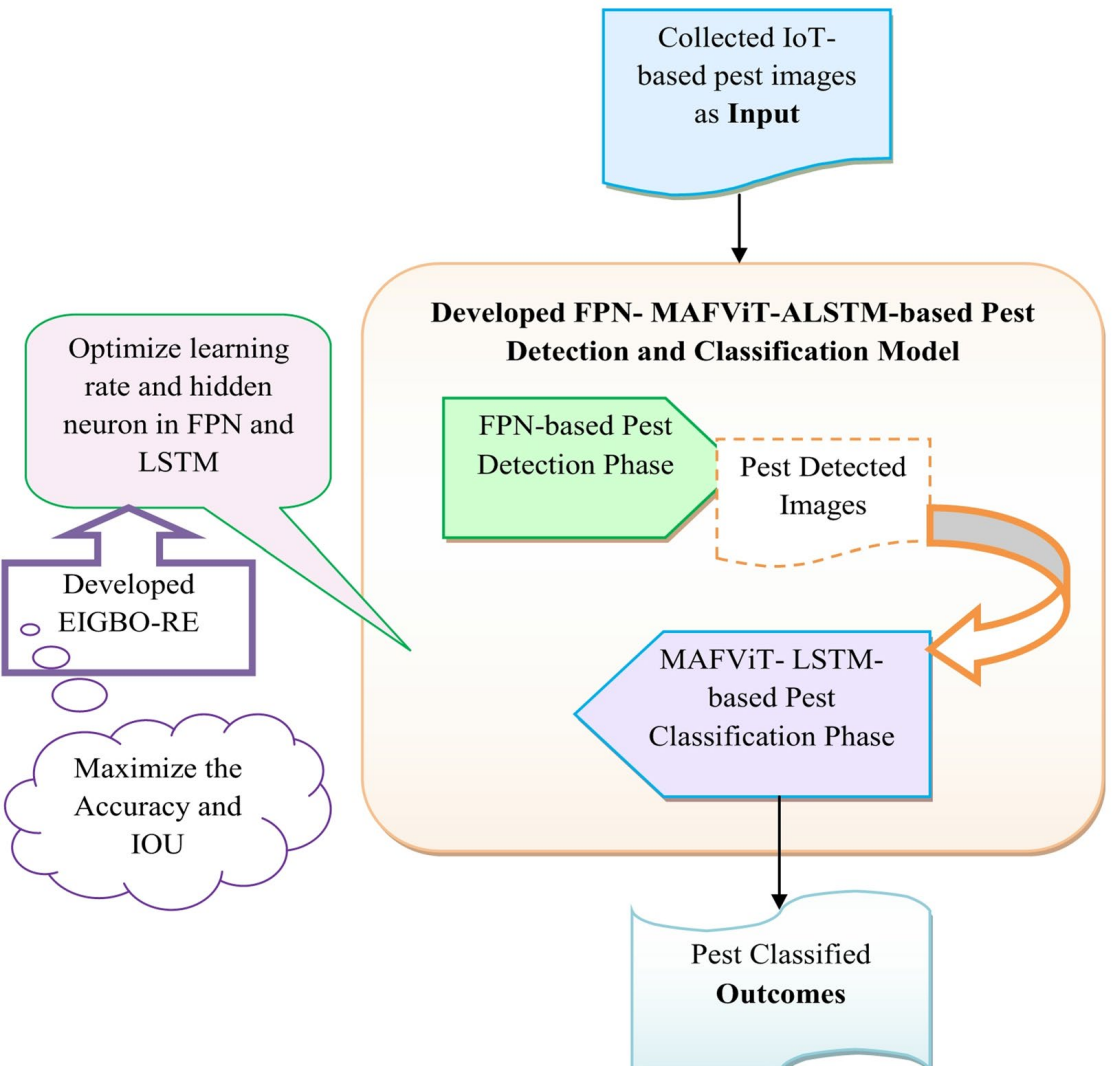
Visual illustraction of the designed pest detection and classification framework.

Overall work flow: In the beginning phase, the essential images are gathered from benchmark resources, which collects the images through IoT sensors. Then, the gathered IoT-based plant images are inputted into the FPN-MAFViT-ALSTM-aided pest detection and classification technique. In this phase, pest detection is carried out using the FPN, and pest classification is performed through MAFViT-ALSTM. In the FPN-based pest detection phase, collected IoT-based images are used as the input. Here, the pyramidal architecture in the FPN excels in analyzing objects with different scales. The pooling layer creates new feature maps with different semantic sizes that can reduce the spatial dimension in the images. Then, the FPN-based detected images are fed into MAFViT-ALSTM for classifying the pests, which is crucial to mitigate potential damage to crops. The MAFViT-ALSTM considered the LSTM as the basic network with MAFViT, and then the parameter tuning procedure is carried out. The MAFViT can capture global patterns within the detected pest, allowing the model to understand a complete representation of the input images, and LSTM is capable of analyzing the historical environmental data, which helps to improve the ability to capture temporal relationships. Hence, to enhance the model’s performance, an advanced EIGBO-RE-based optimization algorithm is used that helps to tune parameters such as hidden neurons and learning rate in FPN and LSTM. Tuning parameters support enhancing the accuracy and IOU to provide better pest classification outcomes. The visual depiction of the implemented model is represented in Fig. 2.

Architecture description: In the implemented pest detection and classification mechansim, required images are collected from an IoT sensor-based image collection on a standard database. Next, the collected images are offered FPN-MAFViT-ALSTM-aided pest detection and classification mechansim. Initially, the pest detection procedures are carried out through FPN. Once the pest detection process is over, then the detected pest images are forwarded to the MAFViT-ALSTM-based pest classification model. In this phase, the LSTM model is considered as the basic network along with it MAFViT is added to attain the significant features. Finally, the pest classified outcomes are obtained from MAFViT-ALSTM. Enhancing the performance of pest detection and classification, parameters in LSTM and FPN are optimized through EIGBO-RE that enhances the accuracy and IoU.

### Technical innovations of the proposed approach

The proposed approach effectively performs pest detection by using advanced deep learning architectural components and a specialized optimization technique, which is helpful to provide reliable performance in terms of higher detection accuracy and robustness.

Multi-attention fusion vision transformer: Traditional CNN approaches struggle to capturet the long-range dependencies and hencet the ViT with a multi-head attention model is integrated to process the global contextual information across the images. This integrated mechanism is helpful to identify the small pests from diverse environmental scenarios.

Integration of feature pyramid network: The Feature Pyramid Network is integrated in the proposed FPN-MAFViT-ALSTM model for generating semantically strong and high-resolution feature maps at multiple scales to detect the varying-sized pests. The extracted multi-scale features are fused using the Multi-Attention Fusion Vision Transformer, which is the novel aspect of the proposed method, ensuring the enhanced global contextual information of the multi-scale feature representation. The robustness of the pest detection mechanism is greatly enhanced through this novel aspect.

Adaptive LSTM (ALSTM): Standard LSTMs are effective for processing the sequential information, but our model introduces an adaptive gating mechanism for dynamically adjusting the memory based on the significance of the incoming features, which is helpful to detect the sudden increase in the pest density variations. This adaptive nature improves the accuracy of the pest detection process, ensuring the stability and robustness of the proposed approach.

EIGBO-RE for parameter optimization: The developed EIGBO-RE is effective for providing better hyperparameters by updating the position based on the fitness function. This leads to faster convergence and provides globally suitable model parameters. Hence, the pest detection performance is greatly enhanced through this optimization process.

### Developed EIGBO-RE

Purpose of EIGBO-RE: A novel nature-inspired optimization algorithm called EIGBO-RE has been developed to enhance pest recognition and classification processes. The advanced module of the GBOA algorithm is known as EIGBO-RE. The purpose of this algorithm is to fine-tune the parameters, such as hidden neurons and learning rate in both FPN and LSTM. In essence, the developed algorithm helps to solve complex problems, which allows the model to precise identification of pest disease detection helping the farmers check for pests and lead to a desired optimal solution, thereby achieving greater efficiency.

GBOA^26^: GBOA is a nature-inspired meta-heuristic algorithm specially designed to identify the best possible solution by imitating the idea of natural selection. This iterative-based optimization process is mathematically expressed in Eq. (1).

1 $$M({(Z + h)_j}{(M{d_{water}})_i}) = {S_j}.{o^{th}}.\cos (2\pi h) + Md_{water}^{}{)_i}$$

Here, the terms ${S_j}$indicate the area of the ${i^{th}}$barnacle and ${j^{th}}$ barnacle, *h* is its random number within − 1, 1, $Z + h$ denotes the ${j^{th}}$ barnacle and $M{d_{water}}$ represents the region corresponding to ${i^{th}}$ a candidate. Despite this, based on the premature convergence in the random number, the EIGBO-RE algorithm can get stuck in local optima. To overcome this issue, a random number *h* in Eq. (1) is modified by using Eq. (2).

Novelty: An advanced optimization algorithm called EIGBO-RE is introduced by updating the random number *h* in the GBOA algorithm as shown in Eq. (2). The use of an updated random number lies in its ability to provide a better optimal solution allows the model to explore various better possibilities, helping to achieve high accuracy.

2 $$h = \frac{{Currentfi{t^2}}}{{Worstfi{t^2} + Bestfit + Currentfi{t^2} - Meanfit}}$$

In this case, *h* denotes the modified random number whereas $Currentfit$, $Bestfit$, $Worstfit$ and $Meanfit$ determine the updated current, best, worst and mean fitness values, respectively. In this phase, the fitness concept is employed to enhance the efficiency of network. Using the fitness concept supports improving the network convergence to accomplish optimal outcomes. Moreover, it helps to offer a reliable outcome in the search space. Including fitness concept for improving the random numbers helps to overcome the premature convergence problems in the local optima region.

**Fig. 3:**
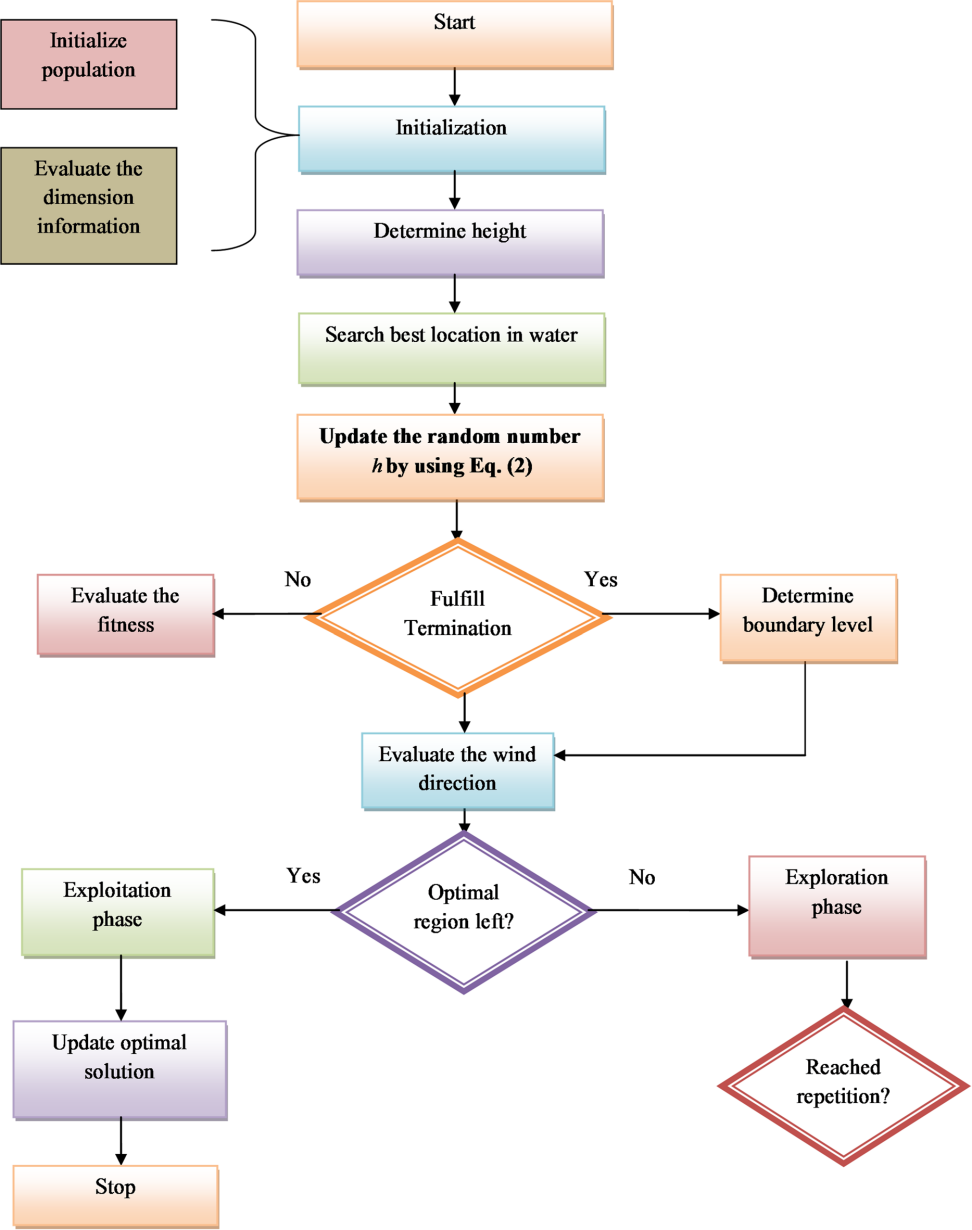
Flow chart of the implemented EIGBO-RE-based optimization algorithm.

Advantages of EIGBO-RE: The developed EIGBO-RE has the potential to enhance the pest detection and classification process by fine-tuning the hyperparameters, such as learning rate and hidden neurons, in the FPN-based pest detection model and the LSTM-based pest classification model. These parameters can help to improve the model performance, thereby reducing overfitting issues. By optimizing these parameters, the designed MAFViT-ALSTM model can understand unseen data, leading to a precise detection process. Particularly in pest detection and classification, the EIGBO-RE algorithm is critical for improving the model’s robustness, which helps to manage resource distribution efficiently. The flowchart of the implemented EIGBO-RE algorithm is provided in Fig. 3.

Flowchart description: Start the EIGBO-RE process. In the initalization phase, population and validation dimension information are updated. Next, the heights are determined, and then the best location in the water is searched. Then, random number updation takes place *h* with a novel fitness-based concept. Furthermore, they are forwarded to the termination condition phase, if the condition is fulfilled, fitness is validated; else boundary levels are determined. Later, the wind direction is validated and moves to the optimal region. If the criteria in optimal regions are fulfilled, then move towards the exploitation phase that supports obtaining the updated optimal solution; else go to the exploration phase that carries out the repetition process. Finally, the overall process of EIGBO-RE has ended.

### Importance of EIGBO-RE-based parameters tuning

Hyperparameter tuning is used for improving the performance and outcomes of the developed MAFViT-ALSTM and its optimization processes. Choosing optimal parameter values can significantly enhance a model’s accuracy and operational efficiency. The proposed EIGBO-RE algorithm offers a smart and flexible way to effective parameter optimization, inspired by the agile movements of a gooseneck. This method effectively reduces the risk of suboptimal problems and increases the probability of identifying optimal solutions. The use of the developed EIGBO-RE algorithm in the pest detection model can reduce overfitting issues. The goal of parameter tuning in pest detection is to balance the model performance while reducing computational cost without compromising accuracy. Therefore, the optimized parameters can help to reduce the errors, thereby preventing overfitting and enhancing accuracy.

## Pest detection and classification using multi-attention fusion and adaptive network

Here, the basic information about the feature pyramid network is given in “Feature pyramid network”. General details associated with LSTM are provided in “Long short-term memory”. Proposed pest detection as well as classification through FPN-MAFViT-ALSTM is highlighted in “Proposed FPN-MAFViT-ALSTM-based pest detection and classification” with their objective functions.

### Feature pyramid network

FPN^27^ provides a significant advantage in locating objects within images. The FPN maintains clarity among larger objects for detection. In ResNeT, when examining the feature layers ranging from D1 to D2, these features map helps to enhance the spatial resolution of rich and high-quality features by up-sampling the semantically rich information as expressed in Eq. (3).

3 $${D_j} = Upsample({D_p}) + {D_h}$$

Here, the term ${D_p}$represents the inferior features ${D_h}$and denotes the superior feature maps.

The multi-scale feature maps extracted from the FPN represent detailed information about the extracted image. The pyramid network extracts feature maps from multiple layers of the backbone. By down-sampling the feature map, the pyramid network captures visual details at different scales.

### Long short-term memory

The LSTM^28^ neural network is a specialized form of the Recurrent Neural Network (RNN) architecture. The memory cells contain three types of gates, forget $(hl)$, input $(tl)$, and output $(kl)$, where *l* represent the current time step. These gates control the flow and unwanted information by updating the cell state $(ml)$, allowing the network to add or discard. The Eq. (4) shows the conversion of temporal data into time-series data.

4 $${h_t} = \sigma ({M_{h,y}}{y_s} + {M_{h,f}}{f_{s - 1}} + {a_h})$$

Here, the term $\sigma$ represents the sigmoid function ${M_{h,y}}$and ${M_{h,f}}$ represents the weight matrices, ${a_h}$ denotes the forge and ${y_s}$is its input and ${f_{s - 1}}$ is its output. The variables needed for compute to process are given by the activation value ${j_s}$ which is shown in Eq. (5).

5 $${j_s} = \tan f({M_{j,y}}{y_s} + {M_{j,f}}{f_{s - 1}} + {a_j})$$

In this case, the bias vectors are denoted as${a_j}$. Thus, the derived cell state is provided in Eq. (6).

6 $${d_s} = {h_s}q{d_{s - 1}} + {j_s}q{\tilde d_s}$$

Here, the terms *q* denote the Hadamard product in each cell state. By implementing a three-gate system, the LSTM network improves the multivariate prediction of disease.

### Proposed FPN-MAFViT-ALSTM-based pest detection and classification

An advanced FPN-MAFViT-ALSTM-aided pest detection and classification scheme is developed in this paper. Here, pest detection is achieved through FPN, and pest classification is performed using MAFViT-ALSTM.

Need for FPN-MAFViT-ALSTM-based pest detection and classification: Presently, various deep learning and machine learning models are being designed for detecting and classifying the pests. Exiting techniques are lacks in robustness and also didn’t validate the noisy information. Understanding the rate of these techniques is very poor, and it also takes more time to adapt in a wide range of applications. Faster-RCNN^18^ is a widely used pest detection model, which is prone to class imbalance issues. Moreover, it failed to handle the abnormalities and also maintain the interpretability also complicated. CNN^19^ always needs enormous samples to execute the training procedure. In some cases, they are prone to overfitting issues, and their implementation expense is higher due to the huge training time. DAMFN^20^ is an efficient mechanism, but it faces certain complications while fusing various features presented in multiple scales. Most of the case, they may lose significant information, which leads to poor efficiency in the validation phase. In order to tackle numerous complications in prior techniques, a new deep learning-aided pest detection and classification technique FPN-MAFViT-ALSTM is designed by determining the strengths of FPN, LSTM and MAFViT along with parameter tuning through EIGBO-RE.

FPN-based pest detection: In this work, an FPN-based pest detection model has been implemented. FPN has been identified as an effective object detection model due to its capability to analyse intricate patterns in the image. The presence of multi-scale feature maps helps to accurately detect even with varying objects.

Novelty of FPN: In the first stage, the gathered IoT-based pest images $P_T^{img}$are fed into the FPN. The FPN is a neural network architecture that leverages the principles of pyramidal architecture with effective feature maps that can help to capture more efficient details in images at variable scales. Once the images are processed via all layers, the processed data are comprised in the form of feature maps. As the sequence of processed data, the feature maps become more effective, which helps to capture high-level conceptual parameters. However, the FPN struggles to capture high-level semantic information effectively due to the uneven contribution of feature maps across different convolutional layers. Therefore, to overcome this challenge, parameters such as learning rate and hidden neurons are optimized in FPN, which effectively enhances the detection process. As the FPN processes more data and identifies complex patterns significantly, it improves the model’s detection capability under various pest species. This benefits ultimately promoting sustainable agricultural practices under varying environmental conditions. Finally, the detected pests are defined by the term $F_E^{img}$.

MAFViT-ALSTM-based pest classification: LSTM is a type of neural network especially developed to enhance the effectiveness of the classification process. By analyzing the images, the designed model may identify the signs of pest infection. The capability of capturing both long-term and short-term memory can help resolve gradient vanishing issues in the network.

**Fig. 4:**
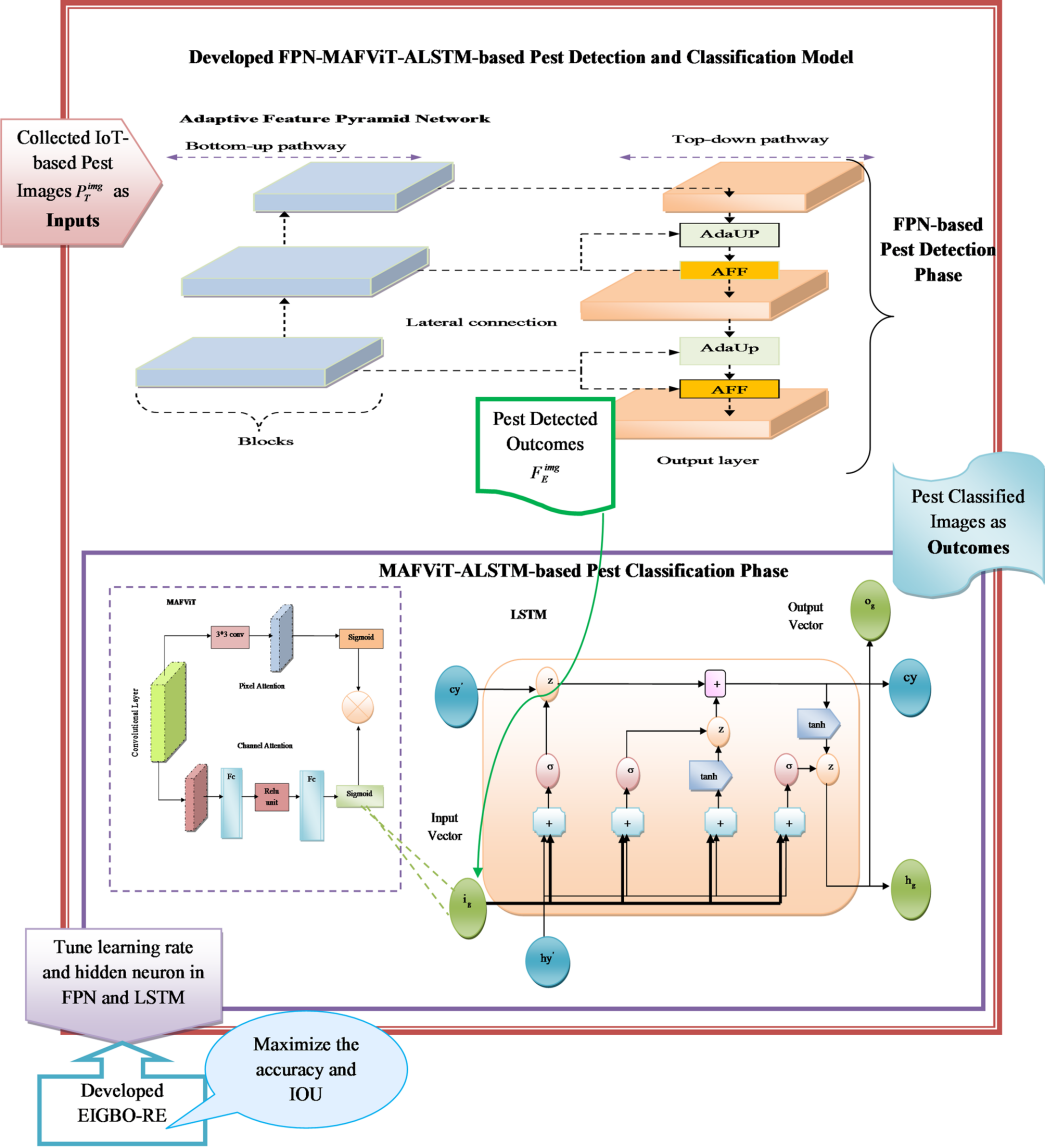
Detailed potrayal of implemented pest detection and classification framework.

Novelty: The procedure starts by uploading the pest-detected images $F_E^{img}$ into the LSTM. This image was processed through the LSTM, which contributes to focuses on the most relevant region in each image. LSTM is good at eliminating unnecessary information. Moreover, the output gate manages the important information to transmit into the current output. Due to the overfitting and high computational complexity, the LSTM makes the process slower. To overcome this issue MAFViT^29^ is added. MAFViT can dynamically adjust the parameters based on the image quality. The ViT allows the elements to capture the most important region of the image by eliminating irrelevant information. This significantly lowers the need for extensive training time and reduces the overfitting issue in the LSTM. The attention mechanism improves the model’s reliability and improves overall performance. By leveraging the principle of a transformer, the LSTM converts the images into a sequence of vectors to understand the relation between the image patterns. When capturing the long-sequence data, the LSTM faces difficulty due to a high vanishing gradient in the convolutional layer. Therefore, to enhance the LSTM function, parameters such as learning rate and hidden neurons are optimized. Hence, the optimized parameters helped to find the optimal solution to handle long-term dependencies. Hence, the strengths of LSTM and MAFViT are considered in this research work, and the designed novel mechanism with parameter tuning is named as MAFViT-ALSTM. This MAFViT-ALSTM approach can effectively classify the pest in the image with high accuracy. The visual representation of the designed strategy is shown in Fig. 4.

Architectural Description of FPN-MAFViT-ALSTM: In this research work, a new pest detection and classification mechansim FPN-MAFViT-ALSTM is employed. The developed FPN-MAFViT-ALSTM is capable of executing two different tasks, such detecting alon with classifying the pests. In the initial phase, the pest detection process is carried out through FPN, and then the pest classification procedure takes place using MAFViT-ALSTM. Initially, the collected IoT-based pest images $P_T^{img}$are given as the inputs to the FPN-MAFViT-ALSTM. Here, the input images are passed to the FPN-based pest detection phase. Here, it includes several blocks to process the samples with lateral connection. In the Adaptive Feature Upsampling (AdaUp) phase, features are tuned optimally through EIGBO-RE and forwarded to the outcome layer. Next, the pest detected outcomes $F_E^{img}$ are attained from the outcome layer of FPN. Further, the pest detection outcomes $F_E^{img}$are forwarded as the input to the MAFViT-ALSTM-based pest classification phase. In this phase, the pest-detected images are offered to the LSTM, which is connected with an MAFViT. Here, the inputs are processed in various scales, and then features are fused with ViT. Finally, at the outcome layer of LSTM, the pest classified outcomes are obtained. In order to enhance the pest detection and pest classification efficiency parameters in FPN and LSTM, such as learning rate as well as hidden neurons, are tuned through EIGBO-RE, which is good at improving the accuracy and IoU.

Objective function: Main objective is given in Eq. (7).

7 $$J{K_{}} = \mathop {\arg \,\,\,\min }\limits_{\left\{ {P_{eng}^{FP},V_{neu}^{FP}} \right\}} \left[ {\frac{1}{{{{\mathrm{H}}_{\mathrm{j}}}}} + \frac{1}{{{{\mathrm{C}}_{\mathrm{s}}}}}} \right]$$

Here, the terms $JK$, $P_{eng}^{FP}$and $V_{neu}^{FP}$indicate the objective function, learning rate and hidden neuron, respectively, whose ranges are selected between $[5,255]$and$[0.01,0.99]$. Equations (8) and (9) show the expression for accuracy ${{\mathrm{H}}_{\mathrm{j}}}$and Intersection over Union (IOU) ${{\mathrm{C}}_{\mathrm{s}}}$.

8 $${{\mathrm{H}}_{\mathrm{j}}} = \frac{{({A_m}T + {B_g}H)}}{{({A_m}T + {B_g}H + {F_c}D + {I_a}M)}}$$

9 $${{\mathrm{C}}_{\mathrm{s}}} = \frac{{{A_m}T}}{{({A_m}T + {F_c}D + {I_a}M)}}$$

Here, the terms ${A_m}T$,${B_g}H$, ${F_c}D$and ${I_a}M$ represent the true positive, true negative, false positive and false negative, respectively.

Advantages and practical applications: The developed FPN-MAFViT-ALSTM-aided pest detection and classification scheme is better at learning the feature maps under different resolutions and also quickly identifies the small objects. Moreover, it effectively enhances the feature understanding rates in complex classes. Maintains higher border understanding rates without affecting the accuracy. In addition, temporal patterns in the samples are collected and also maintain higher flexibility by tackling the complications. Furthermore, the developed technique maintains robustness under various conditions and also enhances pest detection in complex classes. The developed FPN-MAFViT-ALSTM is good to use in the precise agriculture that supports to perform an automated monitoring process by detecting, classifying and tracking the pest. Moreover, it helps to reduce crop damage and enhance the crop yield. In addition, they are efficient for users under complex conditions and identify the pests in varying scales. The pest detection and classification techniques are also efficient to use in mobile applications and IoT-based sensors for collecting the samples.

## Results and discussion

In this phase, various performance analyses were accomplished in the developed mechanism over prior techniques. The experimental setup is discussed in “Simulation setup”, and different performance measures considered for the validation were elaborated in “Performance measures”. The remaining sections include various experimental validations like K-fold analysis, convergence, ROC, confusion matrix, ablation study and analysis by varying hidden neuron counts.

### Simulation setup

This work was implemented using the high-level programming language called Python. The population size was configured to 10, chromosome length was defined as 4, and the maximum number of iterations was initialized with 50. The effectiveness of the model was benchmarked against various conventional models like Faster-PestNet^18^, PestNet^19^, DAMFN^20^, FPN-MAFViT^33^ and optimization algorithms such as Wombat Optimization Algorithm (WOA)^30^, Kookaburra Optimization Algorithm (KOA)^31^, Lotus Effect Optimization Algorithm (LEOA)^32^ and GBOA^26^. Recent pest detection and classification models used for validation were Dual-Attention Multi-scale Fusion Network (DAMFN)^39^, Transfer Learning with Enhanced Residual BottleNeck Vision Transformer (TL-RBVT)^40^, Self-Activation Optimization Convolutional Neural Network (SAO-CNN)^41^ and YOLOv7 with Cross Stage Partial Network (YOLOv7-CSPNet)^42^.

Details involved in comparative methodology: Different details involved with the developed model while designing were discussed as follows. In this research work, 250 training epochs were considered for the validation. In the training and testing phase, the data samples were divided as 75% data for training and 25% data for testing. In dataset 1, totally 2700 images were available for training 2025 images and 675 images were used. In the 2nd dataset, 4395 images were there. In the training 3296 images and testing 1099 images were considered. Finally, at the 3rd dataset 1530 images were presented, and 1148 images were considered for training, and 381 images were used for testing. Moreover, the hardware and software details have been given in Table 2.

**Table 2:** Hardware and software details.

Software requirements	
Software	Pycharam, version 3.11
	Anaconda, version 3
Hardware requirements	
Machine	Window
Processer	i3
Version	11
Random access memory	8GB
Read-only memory	500GB
Libraries	Matplotlib, keras, operncv-python, numpy, tflearn, prettytable, tensorflow

### Performance measures

The metrics used for evaluating the performance of the developed FPN-MAFViT-ALSTM-based pest detection and classification model are given below.

- Sensitivity is given in Eq. (10).

10 $${B_j} = \,\frac{{{A_m}T}}{{({A_m}T + {I_a}M)}}$$

(b) Precision is defined in Eq. (11).

11 $${J_p} = \,\frac{{{A_m}T}}{{{A_m}T + {F_c}D}}$$

(c) FNR can be provided in Eq. (12).

12 $${P_l} = \,\frac{{{I_a}M}}{{({A_m}T + {I_a}M)}}$$

(d) NPV is calculated by Eq. (13).

13 $${S_g} = \,\frac{{{B_g}H}}{{({B_g}H + {I_a}M)}}$$

(e) Critical Success Index (CSI) is analyzed by Eq. (14).

14 $${C_d} = \,\frac{{{A_m}T}}{{({A_m}T + {I_a}M + {F_c}D)}}$$

(f) Matthews Correlation Coefficient (MCC) is validated by Eq. (15).

15 $${K_j} = \frac{{{A_m}T \times {B_g}H - {F_c}D \times {I_a}M}}{{\sqrt {({A_m}T + {F_c}D)({A_m}T + {I_a}M)({B_g}H + {F_c}D)({B_g}H + {I_a}M)} }}$$

(g) Bookmaker Informedness (BM) can be obtained by Eq. (16).

16 $${Z_d} = \,{B_j} + {G_m}$$

Here, the terms ${B_j}$, ${J_p}$, ${P_l}$, ${S_g}$, ${C_d}$, ${K_j}$, and ${Z_d}$, represent the sensitivity, precision, FNR, NPV, CSI, MCC, and BM, respectively.

### Validation on implemented pest detection and classification approach

**Fig. 5:**
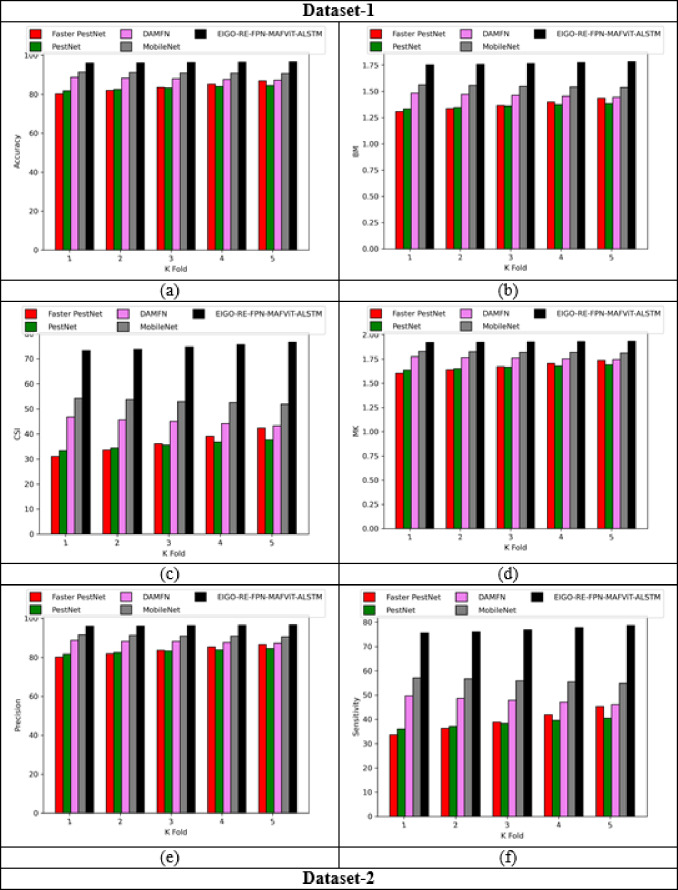

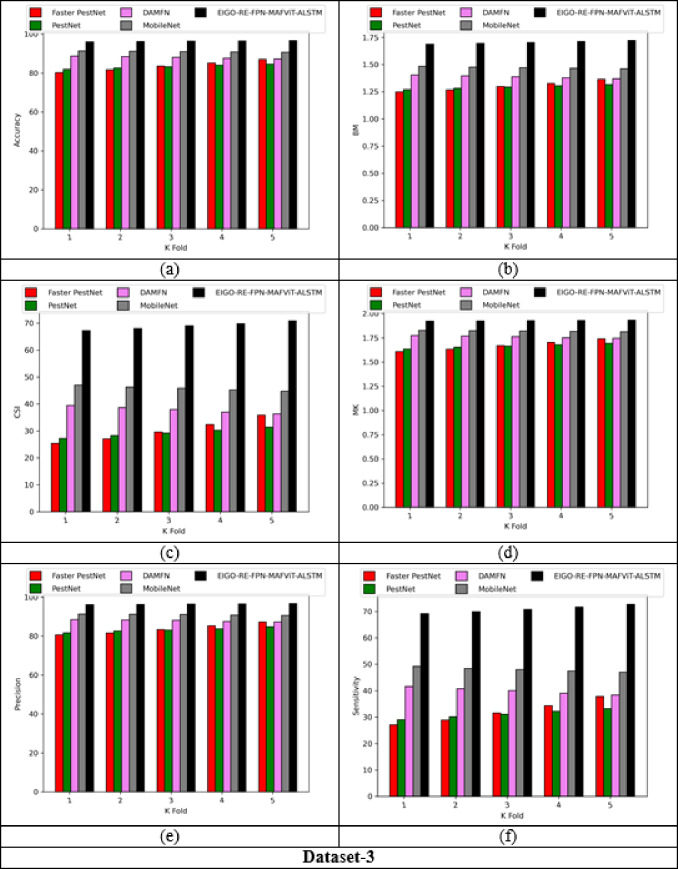

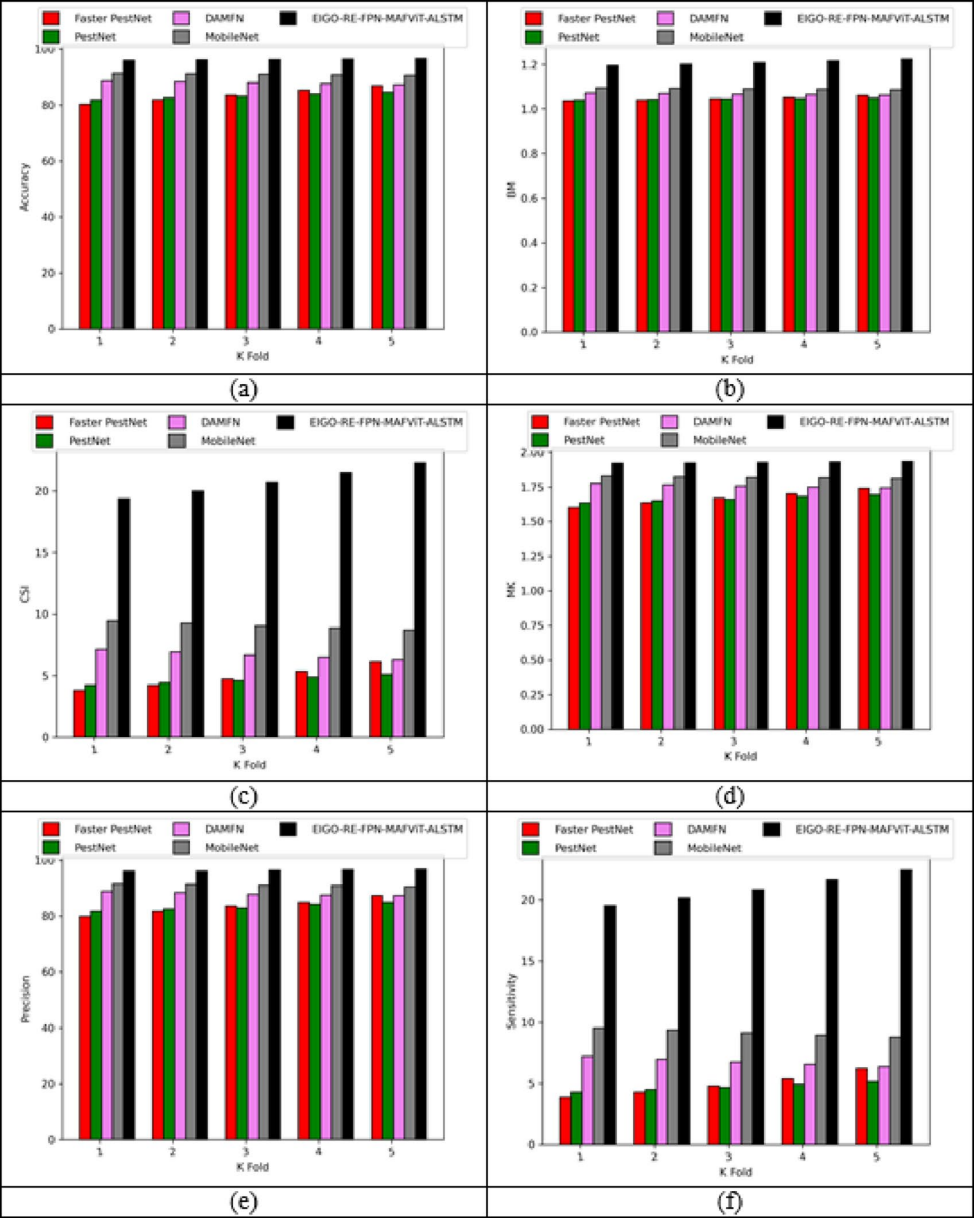
Validation on developed framework over existing models regarding “(**a**) Accuracy, (**b**) BM, (**c**) CSI, (**d**) MCC, (**e**) Precision, and (**f**) Sensitivity”.

**Fig. 6:**
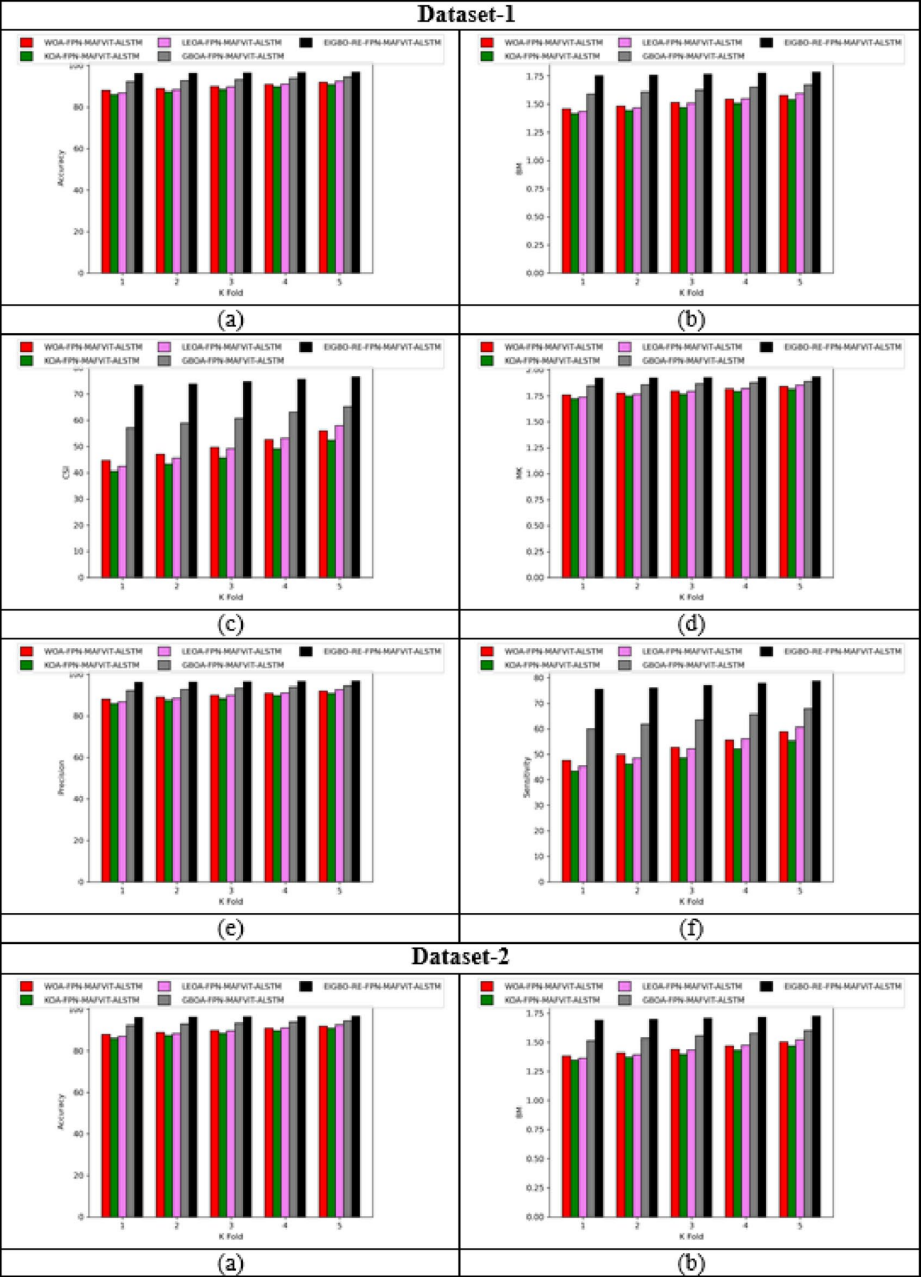

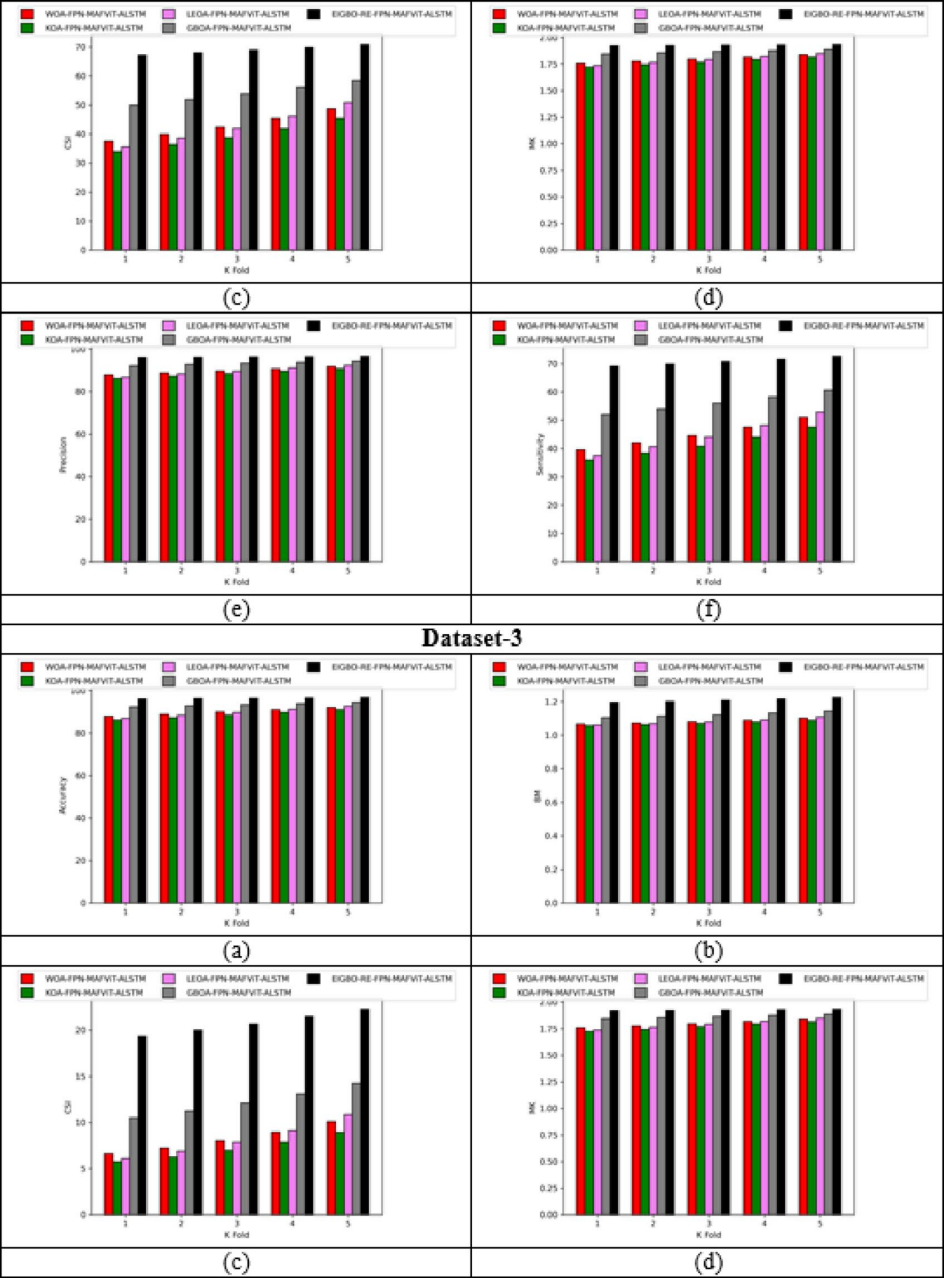

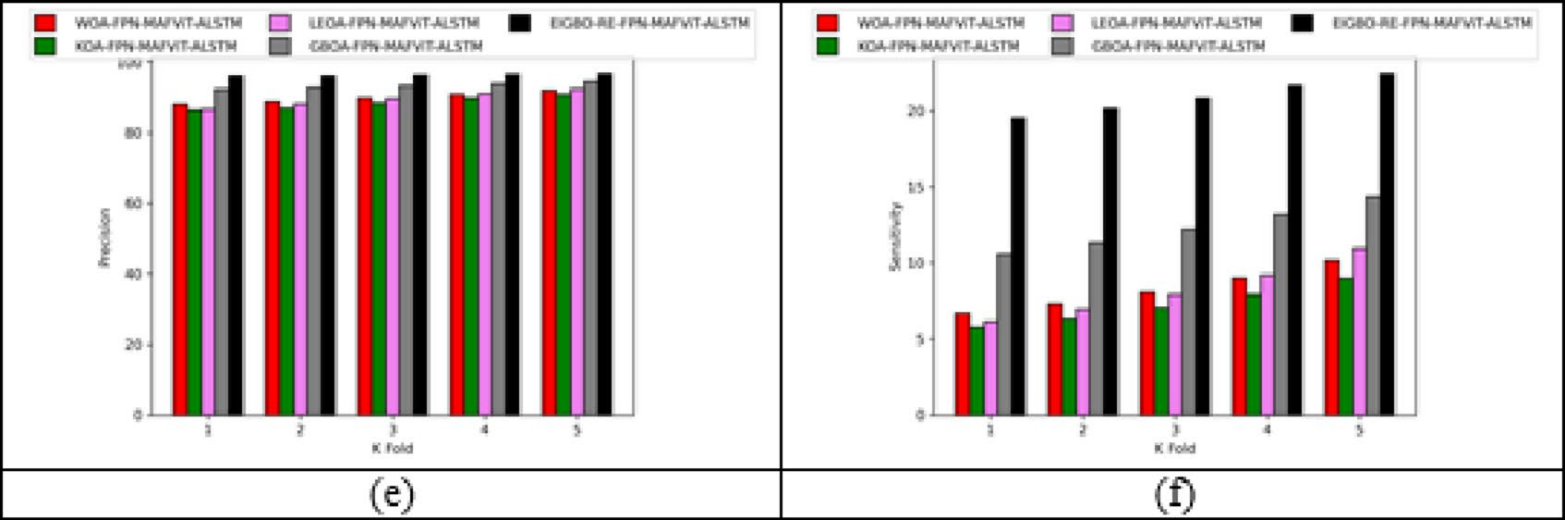
Analysis on designed pest detection and classification scheme for “(**a**) Accuracy, (**b**) BM, (**c**) CSI, (**d**) MCC, (**e**) Precision, and (**f**) Sensitivity”.

Figures 5 and 6 offers the performance validation on designed pest detection and classification mechanism over prior technique. This pest disease detection-based performance analysis is critical in determining the effectiveness and precision of the developed mechanism by comparing it with adaptive algorithms and literature models for detecting pests in the images. Furthermore, the different performance metrics help to optimize detection accuracy and speed, resulting in more efficient pest disease detection processes. The accuracy of the developed FPN-MAFViT-ALSTM in dataset-1 is 22.5%, 19.51%, 8.88% and 5.37% higher than existing models such as Faster-PestNet, PestNet, DAMFN and MobileNet in dataset 1. Improving the accuracy in the developed scheme supports to accomplish superior pest detection outcomes and also reduces the errors. Minimizing the errors in the network improves the overall network performance in different classes. In sensitivity analysis, the developed technique FPN-MAFViT-ALSTM attained a higher efficiency as 78.8% at the 4th fold, and also in precision analysis, 94.3% was achieved at the 1st fold. Thus, the resultant outcomes highlighted the effectiveness of the constructed pest identification model, establishing it as a powerful asset in farming practices. Moreover, the model maintained robustness across different environmental settings, improved pest tracking and timely crop protection action.

### With and without optimization evaluation of designed technique by varying K-fold

**Fig. 7:**
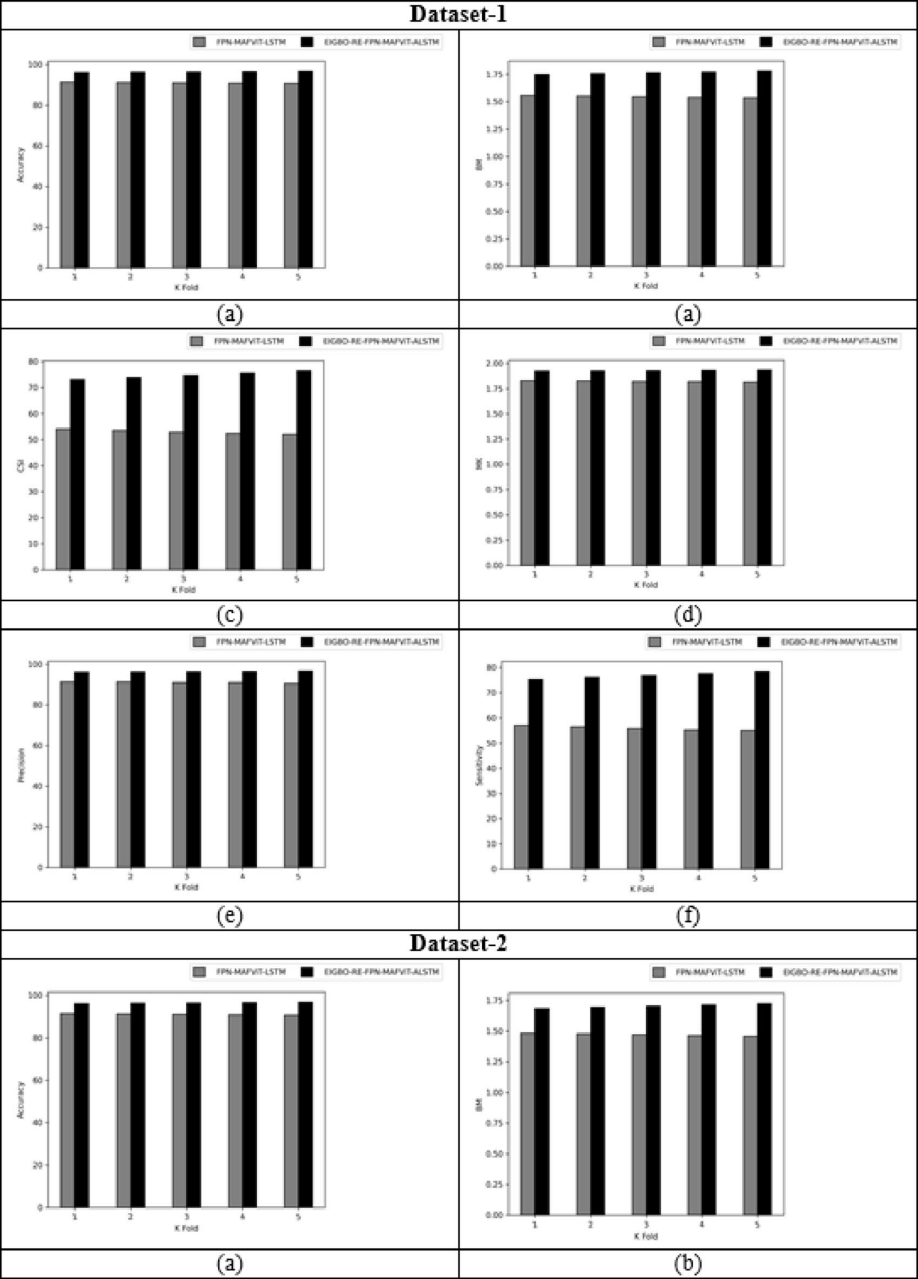

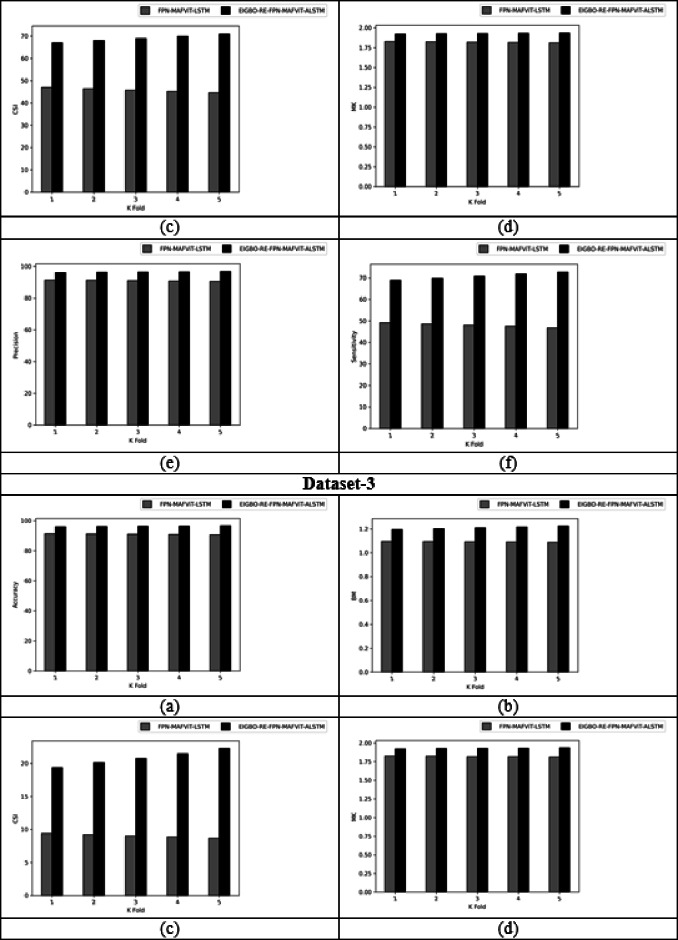

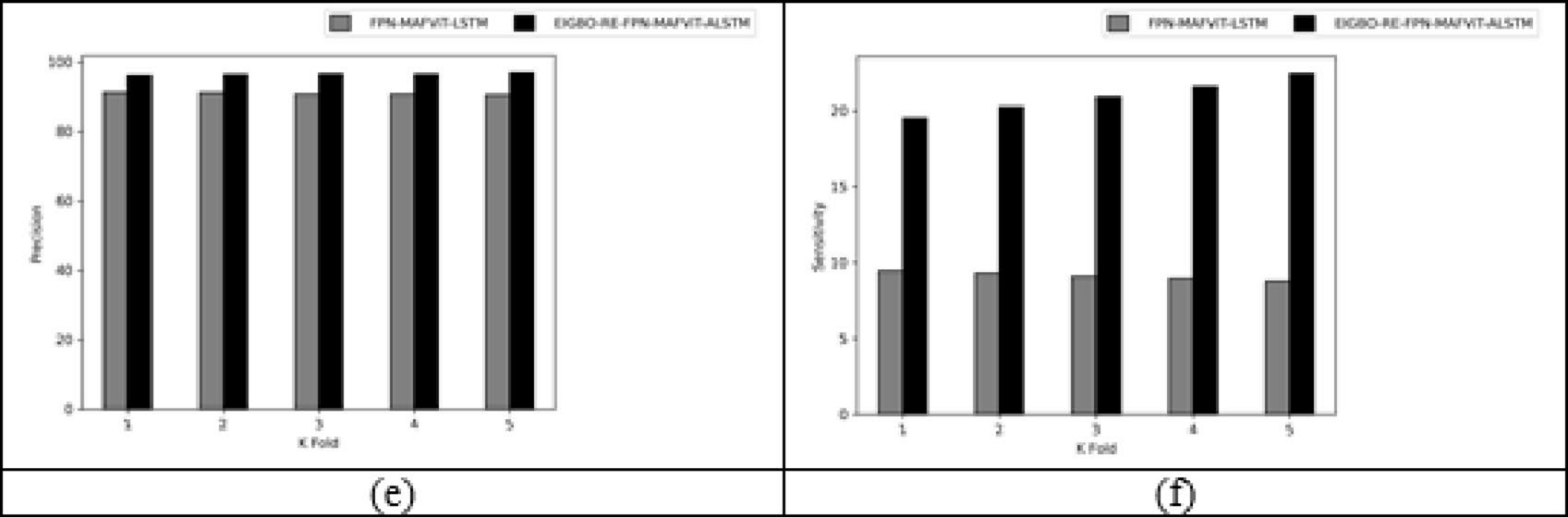
With and without optimization-based evaluation on the designed system by varying K-fold for “(**a**) Accuracy, (**b**) BM, (**c**) CSI, (**d**) MCC, (**e**) Precision, and (**f**) Sensitivity”.

Figure 7 shows the “with and without” optimization evaluation on the developed scheme by varying k-fold. This k-fold evaluation is crucial for estimating the model’s generalization by dividing the dataset based on k-folds. By assessing the model’s parameters using various parameters across diverse datasets, the developed model achieved superior results in terms of prediction accuracy. Moreover, when assigning the k-fold to 2, the proposed FPN-MAFViT-ALSTM model achieved the CSI rate of 6.21% in dataset-2 without optimization. But, the effectiveness of FPN-MAFViT-ALSTM with optimization when setting the k-fold in 3 is 8.88% higher, which shows how well the optimization algorithm optimized the parameters for fine-tuning the model’s accuracy in dataset-1. Sensitivity analysis carried out in the developed EIGBO-RE-FPN-MAFViT-ALSTM-based pest detection and classification model accomplished better performances as 79.1% at the 5th fold, and accomplish 59% without including the optimization scheme at the 1st fold in dataset 1. Here, parameter tuning plays a major role in enhancing the pest detection efficiency of the network. For agricultural professionals, this developed EIGBO-RE-FPN-MAFViT-ALSTM model offers earlier intervention and reduced crop damage by a precisely identified pest.

### Cost function validation on the designed pest detection and classification system

**Fig. 8:**
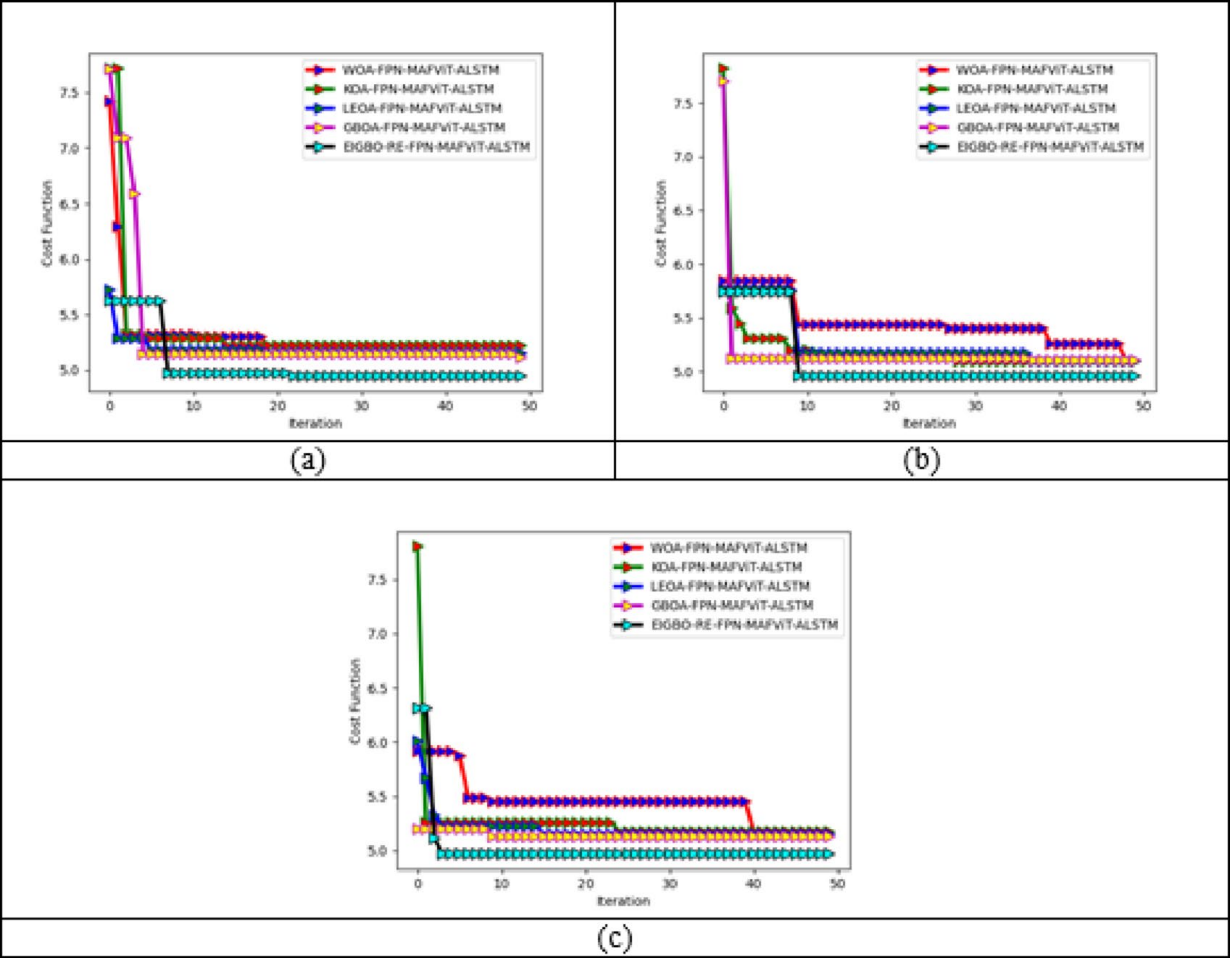
Cost function evaluation of the implemented pest detection and classification mechansim for “(**a**) Dataset-1, (**b**) Dataset-2, and (**c**) Dataset-3”.

Figure 8 illustrates the cost function analysis of the implemented pest identification and classification framework. Cost function analysis is critical in measuring the models performance in order to calculate the financial impact of detection and classification procedures. Here, the validation is carried out over 50 iterations. In the initial stage, the developed model accomplishes slight fluctuations. Later, stable performance is accomplished from the 7th iteration in dataset 1, the 9th iteration in dataset 2 and the 4th iteration in dataset 3. Here, optimal outcomes are accomplished by fulfilling the objective functions. Moreover, it reduces the training process and provides pest detection outcomes quickly without any delay. This assessment analyzes the costs associated with various tests to find the cost-effective way to detect and classify pest diseases. Therefore, it is critical to minimize the operational expenses without compromising its detection performance and accuracy. The estimation considers that the cost reduction should be achieved by minimizing false positives. Consequently, the cost function of the proposed model progressively decreases, which signifies enhanced operational efficiency in the pest detection framework. By facilitating low-cost function, the developed model achieved accurate and early detection of pests with fewer false positives, ultimately helping the agricultural experts to make better decisions while saving input costs.

### Confusion matrix evaluation on implemented framework

**Fig. 9:**
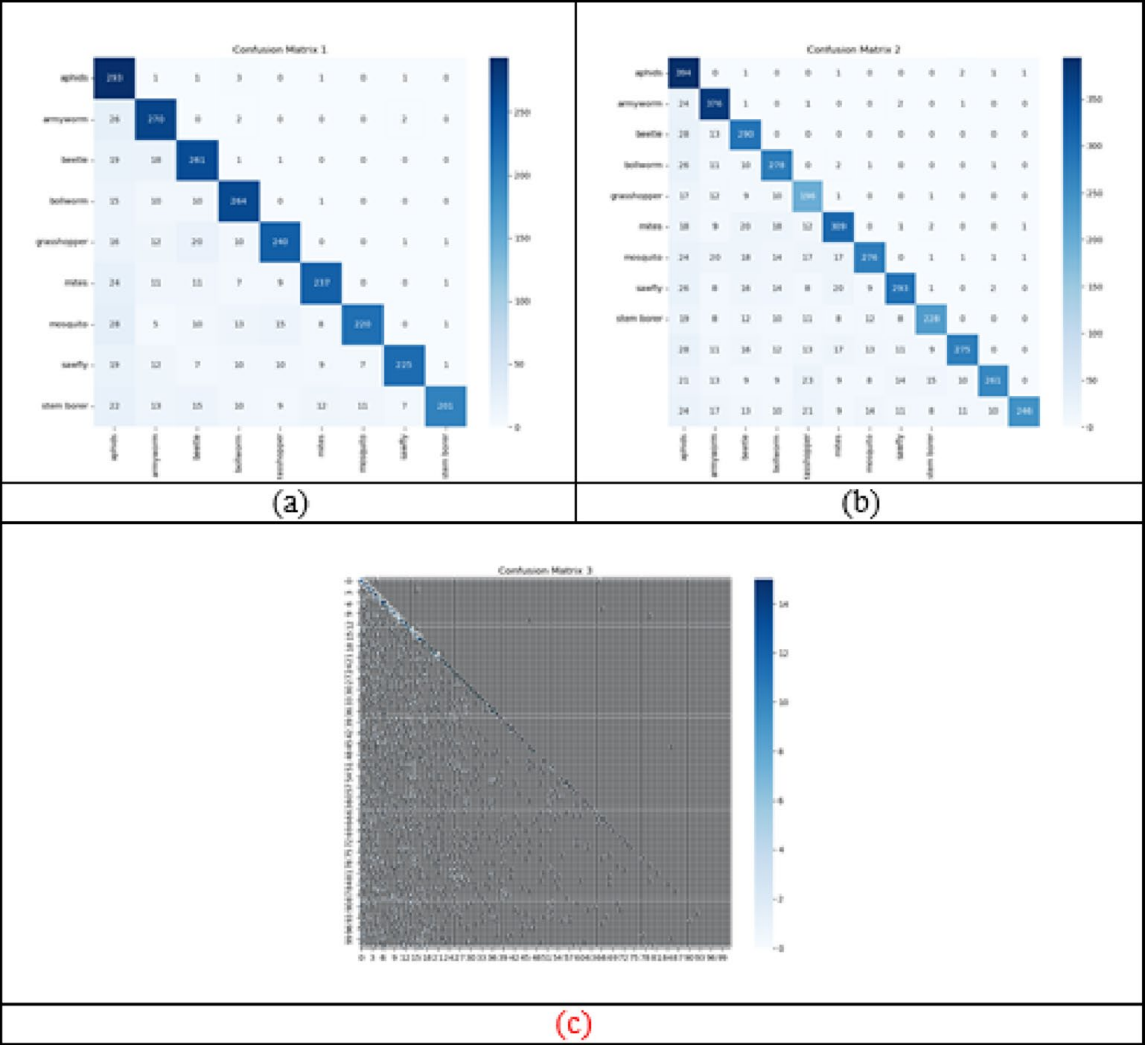
Confusion matrix evaluation on implemented pest detection and classification model for “(**a**) Dataset-1, (**b**) Dataset-2, and (**c**) Dataset-3”.

Confusion matrix validation on implemented pest detection and classification framework is shown in Fig. 9. This analysis is critical for measuring the model’s capability to accurately differentiate between pest-affected and healthy crop instances. By comparing predicted labels with actual outcomes, the confusion matrix offers better results in the model’s classification performance. In order to verify the performance of the developed model, dataset 1 used 9 different classes, dataset 2 includes 12 classes, and dataset 3 has 102 classes. Executing the confusion matrix validation supports to overcome the class imbalance problem that help to accomplish more precise pest classification accuracy. Moreover, it eliminates the overfitting issues that help to demonstrate improved prediction accuracy, lower error rates, and better adaptability to various environmental conditions and pest species. Thus, the findings showcased that the developed pest detection and classification mechanism is better at accomplishing more precise outcomes in different classes.

### ROC curve evaluation on the implemented framework

**Fig. 10:**
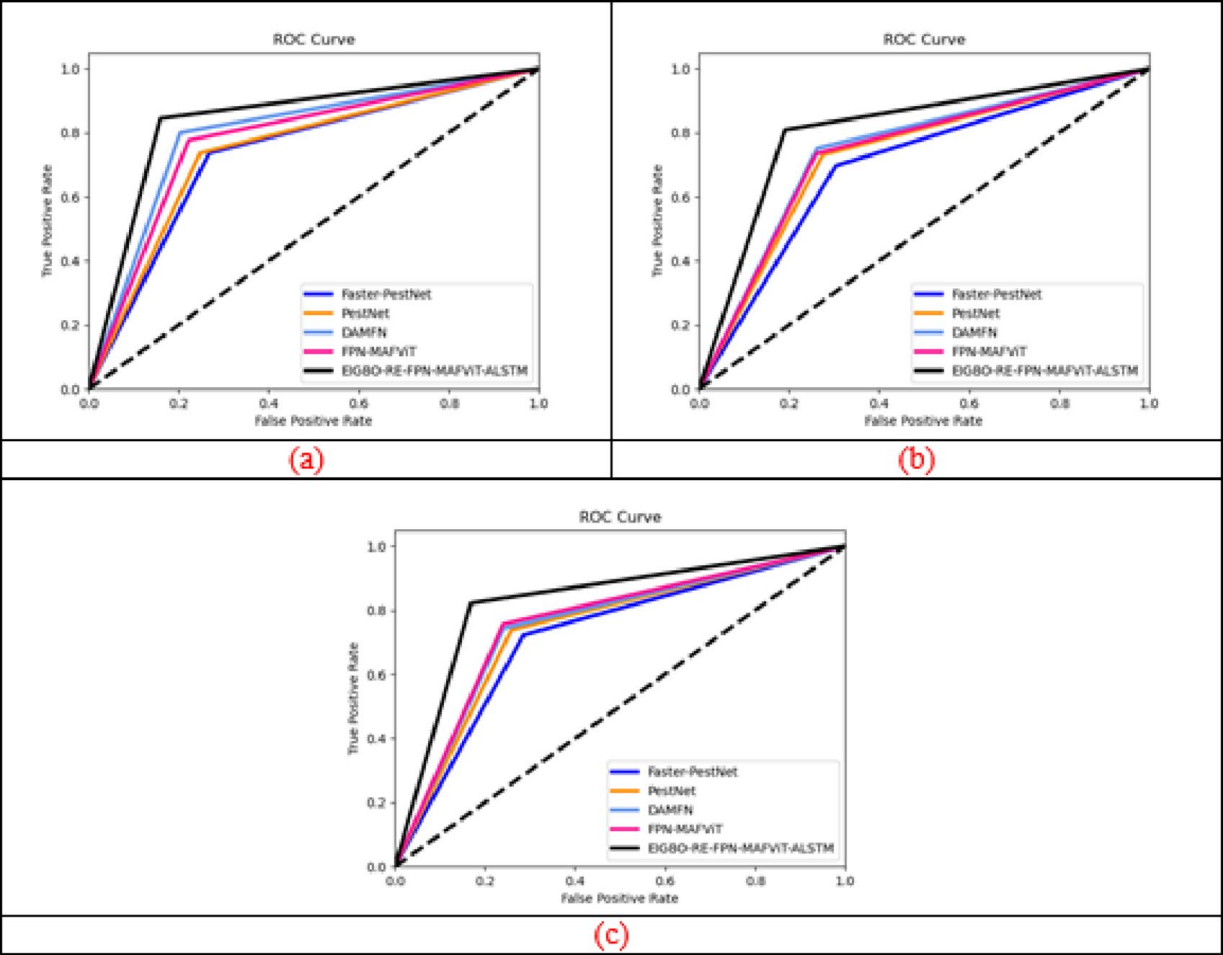
ROC curve evaluation of the developed pest detection and classification approach for “(**a**) Dataset-1, (**b**) Dataset-2, and (**c**) Dataset-3”.

Figure 10 illustrates the ROC curve assessment of designed pest detection framework. ROC analysis is commonly utilized to measure the balance between sensitivity and precision, enabling agricultural experts to evaluate how effectively the system distinguishes between pest-infested and healthy crops. The main goal of this curve is to analyze the trade-off between correct pest detection and minimizing false alarms. Here, validations are carried out over the values true and false positive. Moreover, the threshold is used to vary the performance under different conditions. The ROC validation helps to tackle the invariance issues by selecting the optimal threshold values. Moreover, better balancing between the specificity and sensitivity is managed by selecting the optimal threshold values. In Dataset-3, the proposed FPN-MAFViT-ALSTM model achieved ROC values of 99%, 95.7%, 94.3%, and 92.1%, better outperforming existing models such as Faster-PestNet, PestNet, DAMEN, and FPN-MAFVIT. These results highlighted the developed FPN-MAFViT-ALSTM model’s better performance, robustness, and ability for real-time pest detection in precision agriculture.

### Hidden neuron-based evaluation

Tables 3 and 4 show the varying hidden neuron-based comparison evaluation on the proposed pest identification and categorization strategy. This varying hidden neuron-based comparative analysis is essential for distinguishing how well various optimization algorithms enhance the overall precision of pest detection models. In dataset-1, when assigning the hidden-neuron count in 100, the proposed FPN-MAFViT-ALSTM model outperforms, achieving superior FNR scores of 53.3%, 56.7%, 55.3%, and 38.8%, when compared with existing models like Faster-PestNet, PestNet, DAMFN, and FPN-MAFViT, respectively. This achievement of a higher FNR score emphasizes the efficient performance of the introduced FPN-MAFViT-ALSTM model even across various pest species, thereby minimizing model complexity. In the accuracy validation, the developed EIGBO-RE-FPN-MAFViT-ALSTM accomplished superior performance as 96.72% for dataset 1, 96.68% for dataset 2 and 96.65% for dataset 3 at 500th hidden neuron count. Thus, the analysis outcomes showcased that the developed scheme is better in detecting the pest and classifying the pest type in minimal time. As a result, the introduced model assists farmers in enhancing crop yields and promoting plant growth.

**Table 3:** Varying hidden neuron-based comparison on the implemented approach with traditional algorithms.

Hidden neuron count	WOA-FPN-MAFViT-ALSTM [30]	KOA-FPN-MAFViT-ALSTM [31]	LEOA-FPN-MAFViT-ALSTM [32]	GBOA-FPN-MAFViT-ALSTM [26]	EIGBO-RE-FPN-MAFViT-ALSTM
Dataset-1					
Accuracy					
100	87.914	86.004	86.885	92.313	96.111
200	88.852	87.263	88.313	92.819	96.206
300	89.905	88.358	89.704	93.305	96.383
400	90.893	89.687	91.070	93.881	96.560
500	91.975	90.811	92.514	94.416	96.720
Precision					
100	87.963	85.926	86.815	92.222	96.074
200	88.926	87.481	88.370	92.815	96.148
300	89.852	88.222	89.815	93.407	96.370
400	90.815	89.630	91.000	93.889	96.556
500	91.963	90.741	92.704	94.444	96.704
FNR					
100	52.376	56.562	54.705	39.971	24.439
200	50.094	53.858	51.425	38.230	23.960
300	47.318	51.318	47.872	36.490	23.086
400	44.487	47.912	43.955	34.275	22.179
500	41.105	44.733	39.321	32.127	21.332
NPV					
100	87.907	86.014	86.894	92.324	96.116
200	88.843	87.236	88.306	92.819	96.213
300	89.912	88.375	89.690	93.292	96.384
400	90.903	89.694	91.079	93.880	96.560
500	91.977	90.819	92.491	94.412	96.722
Dataset-2					
Accuracy					
100	87.802	86.028	86.904	92.306	96.104
200	88.857	87.281	88.275	92.825	96.234
300	89.858	88.356	89.681	93.354	96.392
400	90.904	89.634	91.113	93.881	96.528
500	91.983	90.901	92.537	94.429	96.689
Precision					
100	87.918	86.166	86.689	92.241	96.155
200	88.851	87.258	88.373	92.833	96.246
300	89.716	88.464	89.625	93.402	96.428
400	90.739	89.625	91.195	93.857	96.519
500	91.832	90.808	92.423	94.380	96.678
FNR					
100	60.434	64.097	62.396	47.832	30.857
200	57.975	61.585	59.360	45.953	30.094
300	55.396	59.168	55.866	43.921	29.178
400	52.399	55.989	51.757	41.754	28.345
500	48.944	52.409	47.006	39.348	27.355
NPV					
100	87.792	86.015	86.923	92.312	96.099
200	88.857	87.283	88.266	92.824	96.233
300	89.871	88.346	89.687	93.350	96.388
400	90.919	89.635	91.106	93.884	96.529
500	91.997	90.909	92.547	94.434	96.690
Dataset-3					
Accuracy					
100	87.815	86.070	86.910	92.290	96.078
200	88.835	87.209	88.328	92.812	96.228
300	89.886	88.469	89.680	93.354	96.371
400	90.914	89.664	91.103	93.888	96.541
500	91.965	90.902	92.549	94.417	96.654
Precision					
100	88.039	86.405	86.667	92.353	96.078
200	88.824	86.928	88.170	92.810	96.144
300	89.869	88.431	89.673	93.399	96.405
400	90.915	89.869	91.046	93.922	96.536
500	92.026	90.850	92.418	94.510	96.732
FNR					
100	93.325	94.215	93.847	89.399	80.481
200	92.698	93.694	93.040	88.664	79.847
300	91.914	92.943	92.078	87.785	79.178
400	90.986	92.075	90.799	86.795	78.349
500	89.816	91.002	89.061	85.648	77.566
NPV					
100	87.813	86.067	86.912	92.289	96.078
200	88.835	87.212	88.330	92.812	96.229
300	89.886	88.469	89.680	93.354	96.370
400	90.914	89.662	91.104	93.888	96.541
500	91.964	90.903	92.550	94.416	96.689

**Table 4:** Varying hidden-neuron-based comparison evaluation on the designed approach with traditional models.

Hidden neuron count	Faster-PestNet^1^	PestNet^2^	DAMFN^3^	FPN-MAFViT^33^	EIGBO-RE-FPN-MAFViT-ALSTM
Dataset-1					
Accuracy					
100	80.239	81.840	88.786	91.424	96.111
200	82.033	82.527	88.342	91.280	96.206
300	83.527	83.243	88.016	91.008	96.383
400	85.235	83.984	87.671	90.872	96.560
500	86.905	84.477	87.263	90.667	96.720
Precision					
100	80.148	81.704	88.778	91.593	96.074
200	81.889	82.556	88.185	91.296	96.148
300	83.630	83.222	88.185	91.000	96.370
400	85.407	83.778	87.667	91.000	96.556
500	86.667	84.556	87.259	90.593	96.704
FNR					
100	66.345	63.984	50.259	42.887	24.439
200	63.683	62.875	51.359	43.320	23.960
300	61.196	61.695	52.131	44.146	23.086
400	58.073	60.427	52.942	44.562	22.179
500	54.669	59.507	53.867	45.157	21.332
NPV					
100	80.250	81.856	88.787	91.403	96.116
200	82.051	82.523	88.361	91.278	96.213
300	83.514	83.245	87.995	91.009	96.384
400	85.213	84.009	87.671	90.856	96.560
500	86.935	84.468	87.264	90.676	96.722
Dataset-2					
Accuracy					
100	80.254	81.839	88.669	91.413	96.104
200	81.725	82.543	88.337	91.176	96.234
300	83.498	83.185	88.005	91.039	96.392
400	85.155	83.942	87.575	90.859	96.528
500	86.980	84.553	87.230	90.692	96.689
Precision					
100	80.569	81.729	88.578	91.286	96.155
200	81.615	82.685	88.350	91.217	96.246
300	83.436	83.185	88.146	91.081	96.428
400	85.233	83.777	87.531	90.739	96.519
500	87.235	84.733	87.304	90.603	96.678
FNR					
100	72.972	70.955	58.439	50.821	30.857
200	71.112	69.917	59.221	51.565	30.094
300	68.502	68.978	59.975	51.985	29.178
400	65.715	67.809	60.952	52.535	28.345
500	62.189	66.750	61.684	53.031	27.355
NPV					
100	80.225	81.849	88.677	91.424	96.099
200	81.735	82.530	88.336	91.172	96.233
300	83.504	83.185	87.993	91.035	96.388
400	85.148	83.957	87.579	90.870	96.529
500	86.956	84.536	87.223	90.700	96.690
Dataset-3					
Accuracy					
100	80.215	81.874	88.672	91.398	96.078
200	81.885	82.577	88.342	91.229	96.228
300	83.517	83.177	87.971	91.030	96.371
400	85.221	83.874	87.664	90.834	96.541
500	86.938	84.539	87.292	90.675	96.689
Precision					
100	79.869	81.634	88.824	91.569	96.078
200	81.634	82.484	88.301	91.307	96.144
300	83.529	82.810	87.712	90.980	96.405
400	84.967	84.248	87.386	90.850	96.536
500	87.190	84.902	87.190	90.458	96.732
FNR					
100	96.156	95.731	92.797	90.467	80.481
200	95.728	95.522	93.024	90.657	79.847
300	95.222	95.352	93.265	90.873	79.178
400	94.613	95.083	93.444	91.064	78.349
500	93.802	94.845	93.639	91.236	77.566
NPV					
100	80.219	81.876	88.671	91.396	96.078
200	81.888	82.578	88.342	91.228	96.229
300	83.516	83.181	87.973	91.031	96.370
400	85.224	83.870	87.667	90.833	96.541
500	86.935	84.535	87.293	90.677	96.689

### Analysis on the developed mechansim over recent techniques

The suggested FPN-MAFViT-ALSTM model has a range of improvements to the current state-of-the-art approaches mentioned in literature. Our model combines a Feature Pyramid Network (FPN) with a Multi-Scale feature extraction, Multi-Attention Fusion Vision Transformer (MAFViT) with a global-local attention and Adaptive LSTM with a temporal modeling, unlike which uses a dual-track Swin-DAMFN^39^ architecture with a dual-attention multi-scale fusion network to classify pests and diseases. Whereas achieves good accuracy using a focus and fusion, it does not consider temporal dependencies related to the use of IoT sensors, which are essential when pest monitoring is required in real time. In comparison with the one that uses an improved RBVT-Net with transfer learning^40^ to detect apple leaf disease, our solution is even further than transfer learning and detection by considering a hybrid transformer-LSTM architecture and an optimized system using EIGBO-RE. The combination of RBVT-Net and YOLOv7^42^ in is practical in the case of static disease images but does not have the ability to adopt flexible fusion or time modelling capabilities of our framework and is limited to the application in the context of dynamic agricultural IoT application. An improved SAO-CNN with swarm intelligence optimization^41^ suggests to pest detection with UAVs, based on spatial-temporal analysis and swarm-based optimization capability. Although this strategy enhances the deployment of the UAVs, it is based on traditional CNNs and swarm optimization which is not attention-fused and transformer-based as illustrated by our model. The use of MAFViT and EIGBO-RE allows a more robust feature representation and better optimization, obtained accuracy and adaptability, and finally, is YOLOCSP-PEST, which uses a CSP-based variant of YOLO to localize pests and classify them. This algorithm is an excellent localization and classification speed method that lacks sophisticated attention fusion and temporal models. Overall, the proposed framework, FPN-MAFViT-ALSTM, does not only offer better detection and classification accuracy but also allows adaptive learning in IoT-driven applications which is the limitation of the existing models based on the YOLOs. To conclude, the suggested model, FPN-MAFViT-ALSTM, offer more than the current ones in terms of its ability to detect and classify pests in real time and in dynamic agricultural conditions due to its multi-scale features extraction, attention fusion, learning adaptation, and better optimization.

In this phase, various performance analyses are carried out in EIGBO-RE-FPN-MAFViT-ALSTM and it is represented in Table 5. In Dataset 1, the highest values of accuracy, sensitivity, precision, F1-score, and NPV are obtained with the least FNR compared to all the other approaches, which suggests that the proposed model is more successful in recognizing a greater share of pest samples and non-pest samples and produces fewer false alarms. In the case of Dataset 2 and Dataset 3, the same trend is noted: the proposed framework provides better accuracy and F1-score and significantly lower FNR, which shows that its benefits are not confined to any specific dataset. While analyzing the accuracy of developed EIGBO-RE-FPN-MAFViT-ALSTM, it accomplished comparatively higher pest detection efficiency as 5.69%, 2.8%, 4.7% and 0.94% better than the recent techniques such as DAMFN, TL-RBVT, SAO-CNN, and YOLOv7-CSPNet, respectively. On the whole, these findings indicate that the FPN-MAFViT-ALSTM architecture with the EIGbo-RE optimization can produce a more discriminative and robust IoT-based pest detection and classification system than current DAMFN, TL-RBVT, SAO-CNN, and YOLOv7-CSPNet strategies, particularly the ability to detect pests correctly and a limited number of false negatives.

**Table 5:** Validation on implemented pest detection and classification framework over recent techniques.

Performance measures	DAMFN^39^	TL-RBVT^40^	SAO-CNN^41^	YOLOv7-CSPNet^42^	EIGBO-RE-FPN-MAFViT-ALSTM
Dataset 1					
Accuracy	90.25926	94.53909	91.94239	95.10288	96.72
Sensitivity	53.66498	68.41964	58.77899	70.7989	78.5423
Precision	90.2963	94.44444	92	95.18519	96.704
FNR	46.33502	31.58036	41.22101	29.2011	21.332
NPV	90.25463	94.55093	91.93519	95.09259	96.722
Dataset 2					
Accuracy	91.47895	93.97801	92.34736	95.78119	96.689
Sensitivity	49.39197	58.66629	52.31106	67.36556	75.5324
Precision	91.49033	93.87941	92.44596	95.76792	96.678
FNR	50.60803	41.33371	47.68894	32.63444	27.355
NPV	91.47792	93.98697	92.3384	95.7824	96.69
Dataset 3					
Precision	90.13072	92.28758	90.13072	93.13725	96.732
Sensitivity	45. 2197	48.4629	54.1062	57.5643	73.2643
FNR	91.96527	89.22055	91.83686	88.05833	77.566
Accuracy	89.78918	92.4356	89.96219	93.19941	96.654
NPV	89.7858	92.43707	89.96053	93.20003	96.689

### Ablation study on the developed model

This section includes the ablation study carried out in the developed FPN-MAFViT-ALSTM over other pest detection techniques in Table 6. Here, the ablation study is carried out over the accuracy measure. In the study, the developed FPN-MAFViT-ALSTM accomplished higher pest detection accuracy as 95.75% in dataset 1, 94.76% in dataset 2 and 96.69% in dataset 3. Hence, the analysis outcomes displayed that the recommended FPN-MAFViT-ALSTM-based pest detection model detected the pest in complex classes without any misclassifications. So, it is widely suitable to use in a wide range of applications.

**Table 6:** Ablation analysis on developed pest detection and classification model.

Performance measures	LSTM^28^	ViT^43^	ViT-LSTM^44^	FPN-MALSTM^45^	FPN-MAFViT^46^	FPN-MAFViT-ALSTM
Dataset 1						
Accuracy	92.81	92.53	93.09	93.69	95.07	95.75
Dataset 2						
Accuracy	92.18	92.38	93.18	93.3	94	94.76
Dataset 3						
Accuracy	93.1	93.48	93.85	94.61	95.59	96.69

### Statistical testing on the developed model

Statistical testing carried out in the developed FPN-MAFViT-ALSTM-aided pest detection and classification model is displayed in Table 7. Here, the Friedman Aligned Ranks test is considered to verify the performance of the developed model. In this phase, the significance level is set as 0.005 to verify the performance. Here, the validations are carried out in repeated measures without considering any interval data. This validation supports verifying multiple differences and also helps to maintain the robustness over different classes.

**Table 7:** Statistical testing on developed pest detection and classification model.

Comparison techniques	Statistic values	Adjusted *p*-value	Outcome
Significance value (0.005)			
Faster-PestNet^18^ Vs EIGBO-RE-FPN-MAFViT-ALSTM	1.78885	0.73638	H0 is accepted
DAMFN^20^ vs. Faster-PestNet^18^	1.34164	1	
PestNet^19^ vs. EIGBO-RE-FPN-MAFViT-ALSTM	1.34164	1	
DAMFN^20^ vs. PestNet^19^	0.89443	1	
Faster-PestNet^18^ vs. FPN-MAFViT^33^	0.89443	1	
EIGBO-RE-FPN-MAFViT-ALSTM vs. FPN-MAFViT^33^	0.89443	1	
DAMFN^20^ vs. EIGBO-RE-FPN-MAFViT-ALSTM	0.44721	1	
PestNet^19^ vs. Faster-PestNet ^18^	0.44721	1	
PestNet^19^ vs. FPN-MAFViT^33^	0.44721	1	
DAMFN^20^ vs. FPN-MAFViT^33^	0.44721	1	

### Computational analysis on the developed model

Computational validation in the designed EIGBO-RE-FPN-MAFViT-ALSTM is given in Table 8. Here, the need for the computation time and computation space of the developed framework is discussed. In the computation time analysis, the developed EIGBO-RE-FPN-MAFViT-ALSTM is better in processing the samples in minimal time the classical techniques, i.e. 17.3 min for dataset 1, 16.6 min for dataset 2 and 15.9 min for dataset 3. N addition, the computation space analysis carried out in the developed model uses the space as 223 KB for dataset 1,216 KB for dataset 2 and 200KB for dataset 3. Thus, the validation results concluded that the developed scheme needs minimal computation time and space than the prior techniques.

**Table 8:** Computational validation on the designed pest detection and classification mechanism.

Performance measures	WOA-FPN-MAFViT-ALSTM^30^	KOA-FPN-MAFViT-ALSTM^31^	LEOA-FPN-MAFViT-ALSTM^32^	GBOA-FPN-MAFViT-ALSTM^26^	EIGBO-RE-FPN-MAFViT-ALSTM
Dataset 1					
Computation time (Mins)	26.29583	24.45812	22.68281	22.08508	17.38402
Computation space (KB)	296	288	278	276	223
Dataset 2					
Computation time (Mins)	29.16531	28.49599	27.80824	26.32967	16.64722
Computation space (KB)	297	276	260	258	216
Dataset 3					
Computation time (Mins)	29.54219	29.13242	28.58877	27.31638	15.978
Computation space (KB)	280	274	267	251	200

Validation measures	Faster-PestNet^18^	PestNet^19^	DAMFN^20^	FPN-MAFViT^33^	EIGBO-RE-FPN-MAFViT-ALSTM
Dataset 1					
Computation time (Mins)	20.26652	20.06077	19.97633	18.88364	17.38402
Computation space (KB)	264	233	229	226	223
Dataset 2					
Computation time (Mins)	22.35332	22.21871	16.99594	16.66022	16.64722
Computation Space (KB)	248	235	230	229	216
Dataset 3					
Computation time (Mins)	20.15627	19.22486	18.22168	17.97304	15.978
Computation space (KB)	239	212	205	202	200

### Detailed empirical comparison of the proposed model

The detailed empirical comparison of the proposed approach against three benchmark pest-detection datasets is provided in Table 9. The table results show that the proposed EIGBO-RE–FPN-MAFViT-ALSTM framework provides extensive results than models like Faster-PestNet, PestNet, DAMFN, and YOLOv3. On Dataset 1, the proposed method achieves an accuracy of 96.72%, a precision of 96.704%, and an NPV of 96.722%, and the lowest FNR of 21.332%, which indicates the superior performance over pest detection. These results provide strong evidence about the technical advantages of the combined FPN-MAFViT-ALSTM architecture and the EIGBO-RE optimization strategy by visualizing clear improvements in detection accuracy and error reduction compared to existing methods.

**Table 9:** Empirical validation of the proposed model against existing works.

Performance measures	Faster-PestNet^18^	PestNet^18^	DAMFN^19^	YOLOv3^21^	EIGBO-RE-FPN-MAFViT-ALSTM
Dataset 1					
Accuracy	88.142	92.317	90.628	94.218	96.72
Sensitivity	50.284	63.941	55.612	68.103	78.5423
Precision	88.193	92.224	90.731	94.302	96.704
FNR	49.716	36.059	44.388	31.897	21.332
NPV	88.137	92.331	90.619	94.207	96.722
Dataset 2					
Accuracy	89.203	92.742	91.128	94.982	96.689
Sensitivity	46.912	55.103	49.682	64.728	75.5324
Precision	89.216	92.647	91.226	94.968	96.678
FNR	53.088	44.897	50.318	35.272	27.355
NPV	89.198	92.751	91.119	94.983	96.69
Dataset 3					
Precision	88.624	91.012	88.741	92.118	96.732
Sensitivity	42.153	46.804	51.397	54.912	73.2643
FNR	57.847	53.196	48.603	45.088	77.566
Accuracy	87.531	91.204	88.347	92.271	96.654
NPV	87.527	91.209	88.341	92.268	96.689

### Discussions

Scalability in real-time embedded systems: In the IoT-based pest detection and classification mechanism, discussing the scalability of real-time embedded systems is considered as important for the edge devices in farms. Maintaining the scalability in the farm-based real-time edge embedded system is complicated due to different factors like inherent heterogeneity, data management and connectivity. Moreover, accomplishing higher stability with robustness requires better design procedures while implementing in a wide range of applications. Managing the large real-time dataset in edge devices is complicated as they are limited to processing power. In some cases, they require higher bandwidth and also maintaining stability among the enormous edge devices to the centralized servers is difficult. Making real-time decisions is complicated due to poor connectivity among the networks. Variability issues arise in the environments due to different soil types, crop types, and growth stages affect the scalability while designing a novel system under real-world conditions. In some cases, attaining better real-world decisions is complex due to the presence of diverse sensors and their information. These kinds of issues presented in the real-world embedded system affect the pest detection and classification efficiency in a wide range of classes.

Significance of results in developed model: In this research work, various analyses are considered to verify the overall network performance under different conditions. Here, the K-fold analyses are carried out in the developed EIGBO-RE-FPN-MAFViT-ALSTM. Here, the samples are split and analyzed over different folds. In this validation, the developed scheme maintains higher accuracy of pest detection along with its robustness. Further, the cost function validation on developed techniques is verified over iterations, which achieves comparatively higher efficiency by fulfilling various objectives related with the developed scheme. In this phase, the developed EIGBO-RE-FPN-MAFViT-ALSTM gained superior outcomes by improving the accuracy and IOU, which makes the pest detection process easier and quickly identifies the pests in the complex classes. Moreover, the confusion matrix validations are carried out to verify the false positives and reduce the errors. Then, the ROC curve validation is suggested in the developed model, and it identifies the optimal threshold value to accomplish more precise pest detection and classification outcomes in various classes. Next, a deep analysis is performed by varying the hidden neuron count, which accomplishes superior performance by learning the complicated patterns and also reduces the overfitting issues in the training phase. Next, the efficiency of the developed scheme is verified with the recently designed framework that helps to reduce the bias among the data at the training and testing phases. Further, an ablation study is carried out in the developed scheme that helps to verify the importance of the features and also supports maintaining the trust with higher interpretability over a wide range of classes. Later, the computational time and space validation are performed in the developed scheme, which offers a clear view of the time required for processing the samples. Finally, different analysis carried out in the developed scheme supports to display the overall effectiveness of the EIGBO-RE-FPN-MAFViT-ALSTM in different classes.

## Conclusion

In this study, an advanced IoT-based pest detection and classification mechanism was designed by considering the deep learning technique. Here, the required pest images were sourced from an IoT sensor-based collected dataset. Next, the collected pest images were forwarded to the developed FPN-MAFViT-ALSTM-based pest detection and classification model. At first, the collected data were given to FPN, where the pest detection procedure was carried out. Once the pest was identified in the network, and then they were forwarded to the MAFViT-ALSTM-based pest classification phase. Here, the pest-detected images were analyzed carefully and classified the pests using MAFViT-ALSTM. In order to enhance the pest detection and classification efficiency, parameters such as learning rate and hidden neuron in FPN and MAFViT-ALSTM were tuned through EIGBO-RE. Finally, the effectiveness of the proposed model was assessed under various optimization algorithms and prior models. The accuracy of the designed FPN-MAFViT-ALSTM model in dataset-1 was 22.5%, 19.51%, 8.88% and 5.37% higher when compared with existing models such as Faster-PestNet, PestNet, DAMFN and FPN-MAFViT. Therefore, the resulting outcomes emphasized that the developed approach effectively achieved high precision in detecting pests, allowing farmers to focus on the timely intervention, ultimately enhancing crop production.

Limitations and future scope: However, several difficulties were encountered in the developed model during the classification phase. One of the major issues with deep learning architecture, which leads to high computational complexity, especially when integrating the FPN in different farming systems, and it leads to delays and distributions in the detection process. The data processing across multiple platforms concerns slow processing in real-time response. This challenge will be addressed by implementing standardized protocols across the system. Additionally, the model would need to be enhanced for emerging pest species to achieve a high detection rate on unseen data. In addition, a pre-processing procedure will be considered in upcoming works to eliminate the noise. In some cases, more errors take place in the validation, and these issues will be tackled by including the optimal feature selection procedure that supports to offer better outcomes under different environmental factors without any misclassifications. In upcoming work, the developed framework will be designed by considering the hardware integration that supports to work efficiency in the real-world environment.

## Data Availability

The pest images from IoT-based system were obtained from the website listed below to identify and classify pests.Dataset-1 (“IP102-Dataset”): The images were obtained from the website “https://www.kaggle.com/datasets/rtlmhjbn/ip02-dataset” Access date: 2025-04-21. With a size of 3.19 GB, this file consists of information about 102 classes of pests including rice leaf roller, yellow cutwork, red spider, rice shell pest and so on. The dataset was divided in the range of 6:1:3. This dataset for pest detection provides sustainable information about the pest. Dataset-2 (“Pest Dataset”): The website used to retrieve the pest image is “https://www.kaggle.com/datasets/simranvolunesia/pest-dataset” Access date: 2025-04-21. This dataset classifies crop types, offering valuable information for accurate pest recognition tasks.Dataset-3 (“Agricultural Pest Dataset”): The images are sourced via the website “https://www.kaggle.com/datasets/gauravduttakiit/agricultural-pests-dataset” Access date: 2025-04-21. Using 12 different insect species making it well-suited for training and evaluating models aimed at classifying and identifying insects under different scales.
